# Small nucleolar RNAs controlling rRNA processing in *Trypanosoma brucei*

**DOI:** 10.1093/nar/gky1287

**Published:** 2019-01-03

**Authors:** Vaibhav Chikne, K Shanmugha Rajan, Moran Shalev-Benami, Kathryn Decker, Smadar Cohen-Chalamish, Hava Madmoni, Viplov K Biswas, Sachin Kumar Gupta, Tirza Doniger, Ron Unger, Christian Tschudi, Elisabetta Ullu, Shulamit Michaeli

**Affiliations:** 1 The Mina and Everard Goodman Faculty of Life Sciences and Advanced Materials and Nanotechnology Institute, Bar-Ilan University, Ramat-Gan 52900 Israel; 2 Department of Structural Biology, Weizmann Institute of Science, Rehovot 7610001, Israel; 3 Department of Epidemiology of Microbial Diseases, Yale School of Public Health, New Haven, CT 06536, USA; 4 Department of Internal Medicine, Yale School of Medicine, New Haven, CT 06536, USA

## Abstract

In trypanosomes, in contrast to most eukaryotes, the large subunit (LSU) ribosomal RNA is fragmented into two large and four small ribosomal RNAs (srRNAs) pieces, and this additional processing likely requires trypanosome-specific factors. Here, we examined the role of 10 abundant small nucleolar RNAs (snoRNAs) involved in rRNA processing. We show that each snoRNA involved in LSU processing associates with factors engaged in either early or late biogenesis steps. Five of these snoRNAs interact with the intervening sequences of rRNA precursor, whereas the others only guide rRNA modifications. The function of the snoRNAs was explored by silencing snoRNAs. The data suggest that the LSU rRNA processing events do not correspond to the order of rRNA transcription, and that srRNAs 2, 4 and 6 which are part of LSU are processed before srRNA1. Interestingly, the 6 snoRNAs that affect srRNA1 processing guide modifications on rRNA positions that span locations from the protein exit tunnel to the srRNA1, suggesting that these modifications may serve as check-points preceding the liberation of srRNA1. This study identifies the highest number of snoRNAs so far described that are involved in rRNA processing and/or rRNA folding and highlights their function in the unique trypanosome rRNA maturation events.

## INTRODUCTION

Ribosomal RNA (rRNA) processing in eukaryotes is a complex, multi-step process process that starts in the nucleolus, proceeds in the nucleoplasm and culminates in the cytoplasm (1,2). The process is tightly regulated and involves the recruitment of small nucleolar RNAs (snoRNA) and multiple protein factors, including endo- and exo-nucleases, ATPases, helicases and GTPases. The process begins with a pre-rRNA transcript that includes RNAs destined for both the small subunit (SSU; 18S) and large subunit (5.8S, LSU; 25S/28S in yeast/mammals, respectively) (1,2). These elements are separated by long internally transcribed spacers (ITSs) that are processed during ribosome biogenesis (1,2).

Non-coding RNAs have multiple roles during the different steps of ribosome biogenesis (3). snoRNAs and small nucleolar ribonucleoproteins (snoRNPs) are essential for processing of rRNA and nucleotide modification of the rRNA (4–6). The snoRNAs are classified into two groups, C/D RNAs, which guide 2′-*O*- methylation (Nm) and H/ACA RNAs, which guide pseudouridylation (7,8). Most recently, it was demonstrated that C/D snoRNAs also guide rRNA base acetylation by establishing extended bipartite complementarity around the cytosines targeted for acetylation, similar to the pseudouridylation pocket formation by the H/ACA snoRNPs (9). Five major snoRNAs were reported to function in SSU processing; these include U3, U14 and U17 (snR30), which are conserved in both higher and lower eukaryotes, U22, which is specific to mammalian cells, and snR10 which is specific to yeast (10).

The U3 snoRNP is suggested to be the first to bind the nascent pre-rRNA transcript, near the 5′ end; thus, it is thought to play a crucial role in organizing the active processing complex (11). The U3 snoRNA was shown to base-pair with multiple complementary sites within the 5′ external transcribed spacer (ETS) and the 5′ end of mature 18S rRNA moiety (12–15). Recent Cryo-EM structure analysis revealed that the 90S pre-SSU complex is composed of four modules; the U three proteins (UTP), UTP-A and UTP-B; Mpp10-lmp3-lmp4; Bms1-Rcl1; and the U3 snoRNP. These modules are organized around the 5′ ETS and the partially folded 18S rRNA. The U3 snoRNP is positioned at the center of the 90S particle, stimulating pre-rRNA folding and processing, and locks the pre-ribosomes into an intermediate folding state. This provides the necessary time window for biogenesis events to occur before the release of the 5′ ETS and the subsequent processing steps. Thus, the 90S structure suggests a novel mechanism by which the nascent RNA folds and matures in a protected environment (16–20).

The 60S LSU is composed of 25S, 5.8S and 5S rRNAs and 46 ribosomal proteins. The 25S and 5.8S are divided into six domains that intertwine to form the full structure (21). Processing of the pre-60S subunit requires the mitochondrial RNA processing (MRP) snoRNA that cleaves at the A_3_ site, situated in the ITS1 of pre-rRNA (22). In higher eukaryotes, U8 was shown to function by helping to fold the pre-rRNA, facilitating ITS2 and 3′ ETS cleavage (23). The pre-60S ribosomal particle progressively matures as it translocates from the nucleolus to the nucleoplasm and then to the cytoplasm (24–26). The earliest processing complexes are found in the nucleolus, but ITS2 removal takes place in the nucleoplasm (25). Cryo-EM studies of eukaryotic ribosomes showed that the global architecture of the 60S subunit is first established by domains I, II and VI, which form the solvent shell of the 60S complex. At the early stages of processing, the peptidyl-transferase center (PTC) and the exit tunnel are not assembled. Thus, folding of the solvent side first provides a stable scaffold for generating the mature 60S structure (26–28).

Trypanosomes are parasitic protozoa causing deadly diseases such as African sleeping sickness (*Trypanosoma brucei, T. brucei*), Leishmaniasis (*Leishmania donovani, L. donovani*) and Chagas’ disease or American trypanosomiasis (*Trypanosoma cruzi, T. cruzi*). In addition, this family of organisms serves as an important model system to study the role of post-transcriptional regulation, RNA splicing and RNA editing (29–31). In trypanosomes, rRNA processing is largely distinct from that in yeast and mammals and has an increased complexity since the LSU rRNA is cleaved into two large fragments (LSUα and LSUβ), as well as four discrete small ribosomal RNAs (srRNA or sr) of 70 to 220 nt each (32–35). In the free-living amoeba, *Acanthamoeba castellani*, large rRNA (26S rRNA) is divided into three RNA species (36). Moreover, in insects, 28S LSU rRNA is hydrolytically fragmented into two LSU fragments (37). A similar multiple fragmentation of LSU rRNA also occurs in *Euglena gracilis*, in which the rRNA is fragmented into 16 discrete components (38).

In *T. brucei*, the first cleavage of SSU is mediated by the U3 snoRNP (39,40). Although cleavages within the LSU domain were reported almost three decades ago, relatively little is known about the machinery that mediates these additional cleavages (33,34). Thus, trypanosomes are likely to not only contain snoRNAs found in other eukaryotes, but also trypanosome-specific snoRNA species. Trypanosomes contain an snR30 homolog (41) and also possess an MRP homolog (42). However, bioinformatic searches have failed so far to reveal trypanosome homologs of U14 and U22 (43).

To evaluate the role of snoRNAs in rRNA processing, defects in rRNA maturation were examined under silencing of *Nop1* and *Nop58*, the C/D core proteins (42,43) and *Cbf5*, the H/ACA core protein (41). Our results suggested that both C/D and H/ACA snoRNPs play a functional role in trypanosome-specific rRNA processing. The trypanosome-specific snoRNAs, TB11Cs2C1 and TB11Cs2C2, are required for such specific cleavage events (43). RNA-seq performed on small RNP particles of *T. brucei* identified highly abundant snoRNAs, including TB11Cs2H1 (SLA1), TB11Cs2C3 (snR30), TB11Cs2C1 and TB11Cs2C2 species, in addition to 22 other snoRNAs imbedded within the known snoRNA clusters. Among these, three snoRNAs (TB10Cs4C4, TB6Cs1C3 and TB9Cs2C1) were shown to affect trypanosome-specific processing steps (44). The function of the remainder of these abundant molecules is unknown.

Most recently, it was demonstrated that H/ACA *T. brucei* snoRNAs vary in their abundance (45). Genome-wide mapping of the pseudouridines (Ψ) on rRNA by Psi-seq identified 68 Ψ sites. Interestingly, 21 Ψ sites were shown to be hyper modified in the rRNA of bloodstream parasites, parasitic stage that propagates in the mammalian host. The hyper modified sites are located mainly in Helix 69 and the PTC and are important for the function of ribosomes at elevated temperatures while cycling between the insect and mammalian host. The levels of snoRNAs guiding the hyper modified sites are also elevated in the bloodstream form parasites (45).

Little is also known regarding the protein factors involved in rRNA processing in trypanosomes. The function of several factors, including NOG1, was elucidated, and its silencing lead to defects in ITS2 cleavage, similar to those seen in other eukaryotes (46). The nucleolar RNA-binding proteins NOPP44/46 were shown to function in srRNA1 (sr1) processing (46,47). PUF7 was shown to regulate the processing of LSU (48). In addition, P34/37 were shown to be involved in the transport of 60S ribosomal subunit to the cytoplasm (49). Our previous study identified many of the trypanosome processing factors that are homologous to the yeast proteins (50). It proposed that conserved factors might have additional role in Trypanosome cells.

Recent Cryo-EM studies elucidated the three-dimensional (3D) architecture of *T. brucei* (51), *T. cruzi* (52) and *L. donovani* (53,54) ribosomes at near-atomic resolution, and enabled a detailed view of their highly distinct LSU rRNA organization. The studies indicated that the 5.8S rRNA along with the two large rRNA segments composing the LSU (LSUα and LSUβ) serve as a main rRNA scaffold that shares great similarity with other eukaryotic ribosomes (52–54). The remaining four srRNA segments were found to decorate the main scaffold and are mostly localized to the LSU surface. The studies showed that trypanosome-specific r-protein C-termini extensions tightly interact with the rRNA segments, thereby suggesting their functional role in the overall stabilization of these highly fragmented ribosome species (52). Analysis of the *Leishmania* LSU ribosome structure further indicated that the ends of all the trypanosome rRNA fragments converge into three focal points localized to the LSU solvent-exposed surface (54), suggesting that the unique cleavage events most likely occur following the local rRNA folding, in an order that does not necessarily correlate with the order by which the segments are transcribed. Interestingly, the authors also suggested the presence of 5.8S rRNA in all three-focal points, highlighting the possible role of this chain as a folding center that initiates and controls the LSU rRNA folding events during ribosome biogenesis. Additionally, trypanosome-specific rRNA modifications were found in close proximity to all three focal points and were suggested to provide further stabilization of the fragmented rRNA scaffold by tightening their unraveled ends (54).

In this study, we determined the role of ten abundant snoRNAs by examining how their depletion affects rRNA processing. We show that RNAi silencing of each individual snoRNA resulted in rRNA processing defects that were mostly trypanosome-specific processing events and were characterized by accumulation of rRNA precursors and reduction in the level of distinct srRNA populations. The study reveals that these snoRNAs affect specific cleavage events and are present in distinct processing complexes that are involved in either early or late LSU rRNA processing steps. In agreement with the recent Cryo-EM studies (51–54), our results also suggest that the order of processing events does not correlate with the pre-rRNA transcription and that srRNA1 (sr1) is the first to be transcribed and the last to be processed (52,54). We further show that snoRNAs that do not interact with pre-rRNA sequences also affect sr1 liberation. These snoRNA species guide modifications spanning the region from the protein exit tunnel to sr1 and are localized in close proximity to ribosomal proteins that were shown to be incorporated into the ribosome during late biogenesis steps in yeast (55). Silencing of these snoRNA species reduced the cognate modification of the rRNA and affected the final step of LSU processing (liberation of srRNA1). In this study, we suggest that rRNA modifications may also function as check-points for proper ribosome biogenesis and rRNA folding. Taken together, our study highlights the involvement of 15 abundant snoRNAs in mediating the unique features of trypanosome rRNA processing, which, to our knowledge, is the highest number of snoRNAs ever reported to affect rRNA processing.

## MATERIALS AND METHODS

### Cell growth and transfection

Procyclic *T. brucei* strain 29–13, which carries integrated genes for the T7 polymerase and the tetracycline repressor, was grown in SDM-79 medium supplemented with 10% fetal calf serum in the presence of 50 μg/ml hygromycin and 15 μg/ml G418. Cells were transfected as previously described (56).

### Construction of RNAi constructs

Stem-loop constructs were generated to silence selected snoRNAs, using primers listed in Supplementary Table S1, as described (57). snoRNAi (snoRNA interference) against individual C/D snoRNA species were established using the Gateway^®^ recombination cloning system (58) with minor modifications. Initially, mature snoRNA was cloned in pGEM-T easy vector (Promega) using the primers described in Supplementary Table S1. Upon confirming the insert sequence, the snoRNA-pGEM vector was used as template for a polymerase chain reacion (PCR) using the primers Forward-AAATCTAGAGACGGCCAGTGAATTGTAA, Reverse –ATAACGCGTCCATGATTACGCCAAGCTAT. This PCR product was later cloned into pCR^®^8/GW/TOPO^®^ vector (Invitrogen) and subjected to LR-recombination with the pTrypRNAiGate vector (32) resulting in a snoRNA stem loop construct. The constructs expressing dsRNA were linearized with EcoRV. The expression of dsRNA was induced using 8 μg/ml tetracycline.

### Tagging of TIF6 using CRISPR-Cas9

For the expression of Cas9 in *T. brucei*, a plasmid (Cas9-1NLS-CerFP) was generated by cloning *Streptococcus pyogenes* Cas9 from plasmid pX330 (a gift from Dr Ayal Hendel, Bar Ilan University, Israel) into plasmid SK91-NLS-CerFP (a gift from Dr Susanne Kramer, University of Wuerzburg, Germany). This plasmid was transfected into procyclic 1313 *T. brucei* cells (a gift from Prof. Christine Clayton, ZMBH, University of Heidelberg, Germany). For gene tagging, PPOT v4 plasmid (a gift from Dr Samuel Dean, University of Oxford, UK) was used as a template for PCR amplification, adding coding sequences for eYFP and hygromycin resistance. *Tif6* flanking sequences (Tb11.v5.0246) were amplified using specific primers as described in LeishGEdit (59). The primers are listed in Supplementary Table S1. gRNA was synthesized *in vitro* using T7 polymerase. Cas9 expression was induced 12 h prior to transfection with gRNA and the *Tif6*-specific PCR product, and the transfected cells were cloned (57).

### Primer extension and northern analyses

Primer extension was performed as previously described (56). For northern analysis, total RNA was extracted, separated on agarose-formaldehyde gel or 10% polyacrylamide denaturing gel and analyzed using RNA probes. Primers are listed in Supplementary Table S1.

### Preparation of small RNome library

Whole cell extracts were prepared from 2 × 10^9^ cells as described (30); after extraction with 0.3M KCl, the ribosomes were removed by centrifugation for 2 h at 33 000 rpm in a Beckman 70.1Ti rotor (150 000 × *g*). RNA extracted from the post-ribosomal supernatant (PRS) was used for library preparation, essentially as described (45) and size selected on a E-Gel EX (Thermo Scientific Ltd). The libraries were sequenced on an Illumina NextSeq machine.

### Mapping RNA-seq reads to the genome

The 36 bp sequence reads obtained from the Illumina Genome Analyzer were first trimmed of Illumina adapters using the FASTX toolkit (http://hannonlab.cshl.edu/fastx_toolkit/) and reads of 15 bases or less were discarded from subsequent analysis. The remaining reads were mapped to the *T. brucei* genome (60) (TriTrypDB-2.5; http://tritrypdb.org/common/downloads/release) using SMALT, v0.7.5 (http://www.sanger.ac.uk/resources/software/smalt/) with the default parameters, allowing non-unique reads to be mapped randomly to their best match in the genome. Raw read counts for each snoRNA were obtained using Multicov from the Bedtools suite, v 2.17.0 (61). For each snoRNA that appears multiple times in the genome, the counts for each genomic location were combined. Reads Per Kilobase per Million (RPKM) was utilized as the quantification method to obtain a measure for the expression of each snoRNA. Next, the reads were imported and visualized in the IGV viewer (62).

### Fractionation of RNPs on sucrose gradient

Sucrose gradient fractionation was performed as described (44). Whole cell extract was prepared from 2 × 10^9^ procyclic *T. brucei* cells and fractionated on a 10–30% (w/v) sucrose gradient at 35 000 rpm for 3 h using a Beckman SW41 rotor at 4°C. RNA and protein from each fraction were analyzed by northern and western analyses.

### 3D structural analysis of modified positions in trypanosome ribosomes

The cryo-EM atomic models of *T. brucei* and *L. donovani* ribosomes (PDB IDs 5T5H and 6AZ3, respectively) were used for the validation of modified residues in the LSU. SSU-modified residues were validated using *L. donovani* SSU model 6AZ1. Figure preparation was performed using PyMol and Chimera. *L. donovani* models and maps used for figure preparation were: 6AZ3 (EMD-7025), and 6AZ1 and correspond to *L. donovani* LSU and SSU, respectively.

### Cell permeabilization and transcript analysis

Nascent transcription run-on analysis was carried out using a permeabilization protocol described by Tschudi and Ullu (63). The only deviation from the published protocol is the composition of the transcription buffer. In brief, ∼2 × 10^8^ cells were washed in transcription buffer (TB; 150 mM sucrose, 40 mM l-glutamic acid monopotassium salt, 12 mM MgCl_2_, 10 mM HEPES-KOH (pH 7.9), 1 mM dithiothreitol (DTT), 10 μg/ml leupeptin [Sigma]) and incubated in 0.4 ml TB containing 0.2 mg lysolecithin (l-α-lysophosphatidylcholine palmitoyl [Sigma]) on ice for 1 min. Non-induced cells, or cells after 3 days of silencing were permeabilized. Transcription was performed in the presence of 100 μg/ml amanitin. Total labeled RNA was extracted from the cells and fractionated on a 6% denaturing gel.

### ‘RNA walk’

UV Cross-linking was performed essentially as described in (64). Briefly, *T. brucei* cells were harvested and resuspended at 5 × 10^7^ cells/ml and washed twice with phosphate- buffered saline (PBS). Cells (∼10^9^) were concentrated and incubated on ice. 4′-Aminomethyl-trioxsalen hydrochloride (AMT) (Sigma) was added to the cells at a concentration of 0.2 mg/ml. Cells treated with AMT were kept on ice and irradiated using an UV lamp at 365 nm at a light intensity of 10 MW/cm^2^ for 30 min. Next, the cells were washed once with PBS and deproteinized by digestion with proteinase K (Roche) (200 μg/ml in 1% sodium dodecyl sulphate for 60 min). RNA was prepared using TRIzol (Sigma) reagent. Approximately 250 μg of RNA was used for affinity selection, essentially as described (64), using anti-sense oligonucleotide (Supplementary Table T1). After affinity selection, the RNA was used for RT-PCR with the indicated primers.

### Chimera analysis of UV cross-linked snoRNA–rRNA species

To determine which, if any, of the cross-linked species resulted from base pairing interactions between snoRNA and the rRNA, procyclic cells (∼10^9^) were incubated with AMT-psoralen, as described above. Total RNA was fragmented, dephosphorylated using alkaline phosphatase and purified. The RNA was then ligated using RNA ligase (Thermo Scientific) at 25°C overnight and again purified, and cross-linking was reversed by irradiation at 254 nm (65). The RNA was recovered and was subjected to RT-PCR with the indicated primers (Supplementary Table T1).

### Mapping of 2′ -*O*-Methylated residues by primer extension

The primer extension analysis for mapping nucleotide 2′-*O*-Methylation (Nm) was performed using either 1 mM final deoxynucleoside triphosphate (dNTP) (high dNTP sample), or 0.004 mM final dNTP (low dNTP sample), exactly as described in (66,67). In this method, the reverse transcriptase (RT) stops 1 nt before the modified base. Primers complementary to region 3′ to the predicted Nm site were used for the primer extension reaction (Supplementary Table S1). Primer extension products were analyzed on an 8% polyacrylamide denaturing gel, alongside DNA sequencing reactions electrophoresed next to the primer extension products using the same primer used in the primer extension assay. Band intensity was quantified using ImageJ software (http://imagej.nih.gov/ij/).

### Mapping the Pseudouridine nucleotides by the CMC- Primer extension method

Total RNA (20 μg) from *T. brucei* cells was treated with 50 μl of CMC (N-cyclohexyl-N′ -β -(4-methylmorpholinium) ethylcarbodiimide p-tosylate) in buffer (0.17 M CMC in 50 mM bicine, pH 8.3, 4 mM EDTA, 7 M urea) at 37°C for 20 min. Following ethanol precipitation, the pellet was dissolved in 80 μl of Na_2_CO_3_ (50mM, pH 10.4) at 37°C for 4 h to remove all CMC groups from Us and Gs. The treated RNA was used as a template in primer extension analysis. In this method, the RT stops 1 nt before the modified base (68,69). Primer extension assay was performed as mentioned above.

## RESULTS

### Identification of *T. brucei* snoRNAs that affect rRNA biogenesis

**Figure 1.**
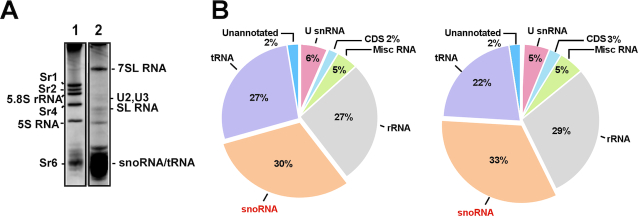
PRS as a source for analyzing the small RNome. (**A**) The pattern of small RNAs present in the PRS. Whole-cell extracts from 2 × 10^9^*Trypanosoma brucei* cells were prepared and depleted of ribosomes, as described in the ‘Materials and Methods’ section. The RNA was extracted from the PRS and separated on a 6% denaturing gel and stained with ethidium bromide. (**1**. Total RNA, **2**. RNA from PRS). (**B**) Pie diagram showing the relative abundance of the snoRNAs. The relative percentage of different RNA species present in the two biologically independent procyclic PRS RNA-seq is indicated.

We previously described the small RNome of *T. brucei*, based on samples derived from RNPs fractionated on an FPLC sizing column (FPLC library) (44). The study identified 140 snoRNAs; among them, 26 molecules were more abundant than the rest of the snoRNA population (Supplementary Table S2). We wished to understand why these snoRNAs are more abundant and whether their abundance reflects their function. To obtain a more comprehensive overview of the small RNome, and verify snoRNA abundance using a different fractionation approach, RNA-seq was performed on RNAs enriched in the PRS (depleted of ribosomes), which should include the entire subset of snoRNAs (Figure 1A). The composition of two biological replicate PRS libraries determined by RNA-seq is presented in Figure 1B. These libraries were highly enriched for snoRNAs, and the data were used to calculate the relative abundance (RPKM) of each snoRNA based on two independent biological replicates (Supplementary Table S2). The RPKM of these PRS RNA libraries were compared to the FPLC library (44) (Supplementary Table S2). Notably, we found that the majority of abundant snoRNAs (RPKM > 15 000) that were previously described by FPLC sizing column RNA seq (44) are indeed abundant in the PRS RNA libraries. However, several snoRNAs that were found to be very abundant in the two PRS libraries were not found at high abundance in the FPLC sizing column, for example, TB10Cs1H3 and TB9Cs3C1 (Supplementary Table S2).

**Figure 2.**
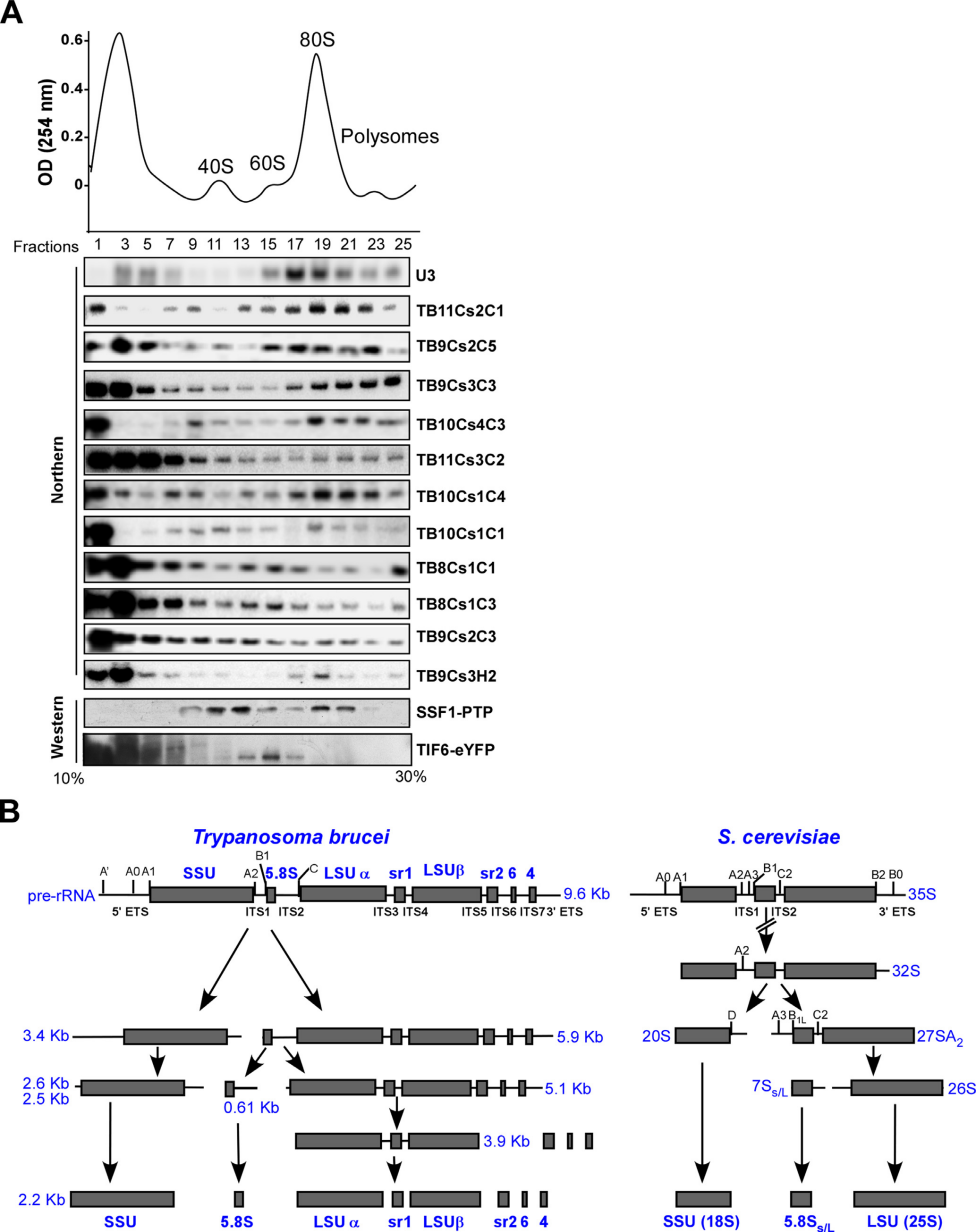
(**A**) Fractionation on a 10–30% sucrose gradients revealed two types of snoRNPs. Whole cell extract was prepared from 2 × 10^9^ procyclic *Trypanosoma brucei* cells and fractionated on sucrose gradient at 35 000 rpm for 3 h using a Beckman SW41 rotor. A total of 400 μl fractions were collected using the ISCO gradient fractionation system. The fractions were deproteinized, and the RNA was separated on a 10% polyacrylamide-denaturing gel and subjected to northern blotting using probes hybridizing to snoRNAs, as indicated. Whole cell extracts were prepared from cells tagged for SSF1*-*PTP and TIF6-eYFP and fractionated by sucrose gradient density centrifugation. The distribution of SSF1 and TIF6 was determined by western blotting using antibody against tag protein. (**B**) Schematic representation of pre-ribosomal RNA processing pathway in *T. brucei and Saccharomyces cerevisiae*. The positions of the major rRNA cleavage sites with ITS and ETS are indicated on top. Below, the different intermediates and products generated from these cleavages during processing are shown.

To gain increased insight into the biochemical properties of the abundant snoRNAs, whole cell extract was fractionated on sucrose gradient, and the fractions were subjected to northern blot analysis. The results (Figure 2A) show that snoRNAs can be grouped based on the size of their cognate RNPs. In the sucrose gradient fractionations (Figure 2A), U3 snoRNA was found in large nucleolar RNP (∼90S, gradient fractions 15–21), which represents the SSU processome. The *Trypanosome*-specific snoRNA, TB11Cs2C1, was shown to be involved in SSU processing (43) and is found in larger complexes of a size similar to the SSU processome. Our data show that most snoRNAs examined here (TB9Cs2C5, TB9Cs3C3, TB10Cs4C3 and TB10Cs1C4), showed a fractionation pattern, like U3 snoRNA (associated with early 90S complexes, gradient fractions 17–23) and a second peak at the top of the gradient (gradient fractions 1–5) that represents the free RNP. A second group of snoRNPs (TB11Cs3C2, TB8Cs1C1, TB8C31C3, TB9Cs2C3, TB9Cs3H2) were present in smaller complexes (gradient fractions 9–15, ∼pre-40/pre-60S complexes), and in fractions 5–7, ranging in size between 20S–30S.

Recent studies characterizing yeast SSU and LSU complexes suggested that complexes involved in early rRNA processing events can be separated from complexes involved in late processing events (70,71). To determine, if the two types of complexes observed in the fractionation (Figure 2A) are likewise involved in different steps along rRNA processing, we examined the fractionation of two different ribosomal factors known to be involved in LSU rRNA processing; SSF1 and TIF6. SSF1 is a factor known to function in early steps of processing, whereas TIF6 functions at later stages (70). The *Ssf1* gene was tagged with the TAP-PTP tag (72) at the C-terminus. *Tif6* was tagged using CRISPR-Cas9 technology (59) by directing YFP to the C-terminus of the gene. The localization of the factors was examined by immunofluorescence verifying their nucleolar localization (Supplementary Figure S1). Extracts were prepared from the tagged cell lines and fractionated on sucrose gradients, and then subjected to western blot analysis. The results shown in Figure 2A demonstrate that the SSF1-containing ribosomal particles migrated with fractions carrying heavy order complexes (fraction 19–21) but were also found in smaller lighter complexes (fraction 11–15). On the other hand, the late factor, TIF6, was found in fractions with smaller complexes in middle of the gradient at pre-60S (fraction 13–15) but also with free RNPs on top of the gradient (fractions 1–7). The results therefore supported the existence of two distinct complexes involved in early or late LSU processing events. The fact that we found most of the TIF6 protein at the top of the sucrose gradient in relatively small complexes (fractions 1–7) suggested that this processing complex may have lost several of its constituents and may represent a breakdown product generated during fractionation. We cannot rule out the possibility that all the snoRNPs present in the pre-40S/pre-60S complexes participate only in LSU processing, since a few of the snoRNAs studied here affect both SSU and LSU processing (see below).

To further investigate the contribution of the different snoRNAs to the distinct rRNA processing steps, the function of the snoRNAs listed in (Figure 2A) was investigated by analyzing their sedimentation with processing complexes on sucrose gradient, *in vivo* crosslinking to rRNA and depletion by snoRNAi. To evaluate the processing defect, it is necessary to inspect the unique processing steps of trypanosome rRNA. A scheme comparing rRNA processing precursors of *T. brucei* to those in yeast is presented in Figure 2B. In *T. brucei*, rRNA processing starts with a 9.6 Kb rRNA precursor which is processed in early biogenesis stages by cleavages in ITS1 to separate the 3.4 Kb SSU precursor (20S in yeast) from the 5.9 Kb LSU precursor (27SA_2_ in yeast). The 5.1 Kb trypanosome LSU precursor (like 26S in yeast) is generated by cleavage in ITS2 to release the 5.8S species from LSU rRNA. However, trypanosomes have unique additional processing steps to remove the srRNAs from 5.1 Kb LSU precursor. A very distinct trypanosome-specific precursor is the 3.9 Kb pre-rRNA species, which separates the large LSU after removal of srRNA2 (sr2), srRNA 4 (sr4) and srRNA 6(sr6). The 3.9 Kb precursor RNA contains the two LSU large fragments and srRNA1 (sr1). All three 5.9 Kb, 5.1 Kb and 3.9 Kb LSU precursors were accumulated in cells defective in rRNA processing upon depletion of NOP1 (42,50).

### The function of snoRNA species interacting with intervening sequences

#### TB9Cs2C5 snoRNA

**Figure 3.**
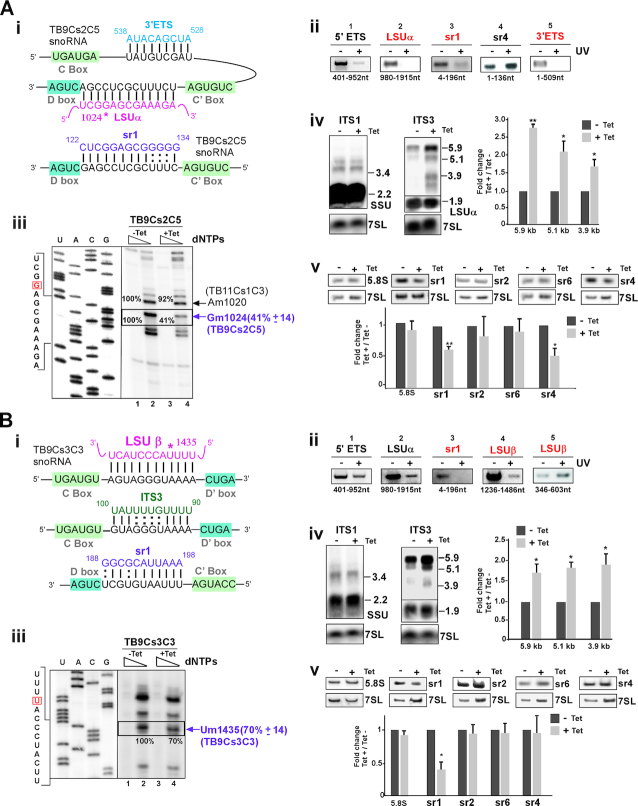
The role of TB9Cs2C5 and TB9Cs3C3 snoRNAs in pre-rRNA processing. (**A**) **TB9Cs2C5 snoRNA** (**i**) The potential base-pair interactions between TB9CS2C5 and pre-rRNA with respect to the C and D boxes. Pre-rRNA and snoRNA sequences are shown with their interactions. The predicted 2′-*O*- methylation site for TB9Cs2C5 snoRNA is marked by an asterisk. (**ii**) ‘RNA walk’ analysis of TB9Cs2C5. Cells were treated with AMT and subjected to UV irradiation; RNA was then subjected to affinity selection with anti-sense biotinylated oligonucleotide complementary to TB9Cs2C5 snoRNA. cDNA was prepared from the affinity-selected RNA using random hexamers and diluted cDNA (1:100) was then amplified by PCR using primers covering the entire rRNA target. The PCR products were separated on a 1 or 1.8% agarose gels and stained with ethidium bromide. RNA from irradiated versus untreated cells is designated (+) and (−), respectively. The domains carrying the cross-linked adducts and primer position is indicated. (**iii**) Validation of reduction in the level of Nm as a result of snoRNA silencing. Total RNA (8 μg) from TB9Cs2C5 snoRNAi cells before (−Tet) or after 3 days of silencing (+Tet) was subjected to primer extension using an oligonucleotide complementary to LSUα region (see Supplementary Table S1 for primers) at low and high-dNTP concentrations (and 0.004 and 1 mM). Extension products were separated on 8% polyacrylamide–7 M urea gels along with a dideoxynucleotide sequence ladder of the LSUα produced using the same primer. Partial DNA sequences are given. The methylated site is indicated by box. Locations of the Nms on rRNA are indicated by arrows at the right. The mean percentage of band intensity corresponding to Nm stop on the rRNA in the TB9Cs2C5 silenced cells (compare lane 2, −Tet to lane 4, +Tet) with the standard deviation based on two independent experiments is given at the right. (**iv**) Accumulation of pre-rRNA precursors following the silencing of TB9Cs2C5 snoRNA. Total RNA (15 μg) was prepared as in (iii) and separated on a 1.2% agarose/formaldehyde gel and transferred to nylon membranes. Northern blots hybridized with the following probes: junction region of SSU-ITS1 (ITS1); junction region of LSU α-ITS3 (ITS3). The sizes of precursors detected are indicated and schematically represented in Figure 2B. The 7SL RNA hybridization was used as a loading control. The precursor size is indicated in Kb. The blots were also hybridized with probes against the intervening sequences of ITS2 and ITS7 (See Supplementary Figure S2A (**v**)). Quantification of pre-rRNA accumulation was determined from the ITS3 northern blot, with the −Tet ratio set to 1. Values represent the means of three independent experiments, and bars represent standard error of the mean. One sample *t*-test was used to determine significant difference from 1.0. *P < 0.05; **P < 0.01. (**v**) Reduction in the level of srRNAs following silencing of TB9Cs2C5. Total RNA (5 μg) was separated on a 10% denaturing gel and subjected to northern analysis with the different srRNA probes. Statistical analysis was performed as in panel A (iv). (**B**) **TB9Cs3C3 snoRNA** (**i**) Model indicating the potential base-pairing interaction between TB9Cs3C3 snoRNA with precursor rRNA. (**ii**) ‘RNA walk’ analysis of TB9Cs3C3. The legends are same as in panel 3A (ii) but anti-sense biotinylated oligonucleotide complementary to TB11Cs3C2 snoRNA was used for the affinity selection. (**iii-v**) The legends are same as for panel 3A (iii-v), but the analysis was performed using RNA isolated from TB9Cs3C3 snoRNAi cell lines that were uninduced (−Tet) or tet-induced (+Tet) for 3 days.

The first snoRNA characterized in this study is TB9Cs2C5, which fractionated with early LSU factors (Figure 2A). The potential interaction of the snoRNA with its targets (Figure 3A (i)) suggested two different sites of interaction, around the distal region (3′ ETS, and LSUα) and around sr1. TB9Cs2C5 snoRNA base-pairing interaction with sr1 is located near 3′ end directly upstream of D box. To investigate if this snoRNA indeed interacts with the three potential sites within LSU rRNA, we treated cells with AMT-Psoralen followed by UV cross-linking and performed ‘RNA walk’ analysis (64). Briefly, RNA derived from cells treated with AMT-psoralen was affinity-selected with anti-sense oligonucleotide to TB9Cs2C5, and the cross-linked adducts were mapped by RT-PCR. Cross-linking between the target RNA and the snoRNA blocks the amplification of the cross-linked domain, and hence no amplification could be detected on RNA extracted from UV treated (+UV) cells (highlighted in red in Figure 3A (ii), compared to control sites that are not predicted to interact by base-paring (Supplementary Figure S2A (i)). The results presented in Figure 3A(ii) suggest that TB9Cs2C5 snoRNA contacts all three predicted domains in the LSU but no other domains in the same RNA. To verify these predicted interaction sites, we used an independent approach based on ligation of snoRNAs to its target RNA, similar to the method recently described (65). Cells were incubated with AMT-Psoralen and cross-linked as described above. After mild fragmentation, the interacting RNAs were ligated; then, the cross-linked adducts were removed by reversal of cross linking using 254 nm irradiation. cDNA was prepared, and PCR was performed with primers derived from both snoRNA and the target rRNA. To examine the validity of this approach, the 5′ ETS interaction with the U3 snoRNA was confirmed based on previous studies (39,40). Our chimera method identified the two expected interactions of U3 snoRNA with the 5′ ETS (highlighted in blue in Supplementary Figure S2A (ii)). After, validating the approach, the interaction of TB9Cs2C5 snoRNA with the its proposed targets on pre-LSU was examined. We detected a strong enrichment for crosslinked RNA duplexes comprised of TB9Cs2C5 snoRNA- ITS3 pre-RNA. Chimeras representing TB9Cs2C5-3′ ETS contacts were also enriched (Supplementary Figure S2A (ii)). Note that the relevant interactions appeared only in the +UV samples, whereas non-specific background interactions appeared regardless of UV cross-linking (−UV) (Supplementary Figure S2A (ii)).

To verify that the modification guided by the snoRNA resulted in reduction in the level of the predicted Nm, Silencing of TB9Cs2C5 snoRNA was attempted using stem-loop RNAi construct which reduce the snoRNA expression after 3 days of tetracycline induction and efficient silencing was observed (Supplementary Figure S2A (iii)). Here we determined 2′-*O* methylated (Nm) nucleotide by primer extension under different dNTP concentration. Nm residues induce a specific reverse transcription (RT) stop one nucleotide downstream of the methylated site under low dNTP (0.004 mM). The results based on two biological independent replicates demonstrate as expected that under low dNTP, the corresponding methylation-dependent stops on rRNA (1 nt before the modification site) at position Gm102 guided by TB9Cs2C5 was specifically reduced upon silencing. (Figure 3A (iii)) However, under TB9Cs2C5 silencing only the Gm1024 but not the Am1020 modification guided by another snoRNA was affected, indicating the specific effect of the snoRNA silencing. We next tested whether this snoRNAs also guides Gm126 on srRNA1. Our mapping data failed to detect the predicted site (Supplementary Figure S2A (iv)) and thus the interaction between the snoRNA and sr1 is likely to be essential for rRNA processing (see below).

To explore the processing defects resulting from the depletion of TB9Cs2C5 snoRNA, northern blot analysis of RNA extracted from un-induced and tetracycline induced cells was performed. The ITS1 probe was designed to hybridize with the junction region between mature SSU and ITS1 to monitor 3.4 Kb pre-SSU precursor. Probing with ITS2 could only detect the 5.9 Kb precursor. The ITS3 probe directed against the junction region between mature LSUα and ITS3 to identify three pre-LSU precursors, 5.9, 5.1 and 3.9 Kb, as well as 1.9 Kb mature LSU α (see scheme in Figure 2B). The ITS7 probe should detect the 5.9 and 5.1 Kb precursors. The results of northern hybridization indicated accumulation of all the three LSU pre-rRNA transcripts (5.9, 5.1, 3.9 Kb) (Figure 3A (iv) and Supplementary Figure S2A (v)). The inhibition of processing should reduce the level of downstream mature srRNAs which are cleaved from the pre-LSU. To confirm this effect, RNA was analyzed by northern blotting on polyacrylamide denaturing gel and hybridized with all srRNA probes. The results clearly suggest reduction in the level of sr1 and sr4, indicating that processing of sr2 and sr6 is not linked to sr4 processing (Figure 3A (v)). The data also further supported a cross-talk between the processing of sr4 and sr1, as was proposed by Liu *et al.* (52). Interestingly, the interaction of the snoRNA with the 3′ ETS region near the D’ box is likely important for liberating the sr4. However, since this D’ box is degenerate, it is possible that it does not bind to NOP56 but this snoRNA domain is involved in base-pairing with the 3′ ETS during processing.

#### TB9Cs3C3 snoRNA

Distribution of TB9Cs3C3 snoRNA on a sucrose gradient showed that this RNA is associated with large pre-ribosomal complexes (Figure 2A). Potential interaction to pre-rRNA was suggested for this snoRNA, which contains three sequence complementarities with ITS3, sr1 and LSUβ (Figure 3B (i)). Using ‘RNA walk’, the TB9Cs3C3 snoRNA association with the predicted sites on sr1 and LSU β were verified (Figure 3B (ii) and Supplementary Figure S2B (i)). Next, the interactions were also examined for the presence of the predicted chimera between the snoRNA and the pre-rRNA target. Indeed, a specific interaction between TB9Cs3C3 snoRNA and ITS3 was detected (Supplementary Figure S2B (ii)). Taken together, with the results from RNA walk analysis, these observations further highlight that chimera ligation method enriched the *in vivo* snoRNA-ITS3 precursor rRNA interaction. To assess the impact of TB9Cs3C3 snoRNA silencing on Nm, and to investigate its function in pre-rRNA processing, the snoRNA was silenced and silencing was verified by northern blotting (Supplementary Figure S2B (iii)). The silencing of the predicted Nm (Um1435 on LSUβ) was examined by primer extension under low dNTP, and the results demonstrate reduction in the level of the specific Nm under silencing of TB9Cs3C3 snoRNA. (Figure 3B (iii)). D box of TB9Cs3C3 snoRNA encompasses 9 bp complementary to the sr1 with substantial AU pairs in RNA duplex.

We next analyzed whether TB9Cs3C3 snoRNA also possesses the potential to guide Nm at C192 on sr1 but no methylation was observed (data not shown). Previously it has shown that minimum 10 bp duplex around the modification site modify target site efficiently (73). To identify if TB9Cs3C3 snoRNA is involved in pre-rRNA processing, the presence of rRNA precursors was examined by northern blotting and accumulation of all 5.9, 5.1 and 3.9 Kb LSU precursors was observed (Figure 3B (iv)). Hybridization with probe ITS2 and ITS7 region produced similar pattern (Supplementary Figure S2B (iv)). TB9Cs3C3 snoRNA silencing also resulted in a specific decrease in the level of mature sr1 (Figure 3B (v)). This snoRNA seems to also have a dual function, since it both guides a modification and also directly affects rRNA processing. Together, these results indicate that the TB9Cs3C3 is involved in both early and late LSU rRNA processing events.

#### TB10Cs4C3 snoRNA

**Figure 4.**
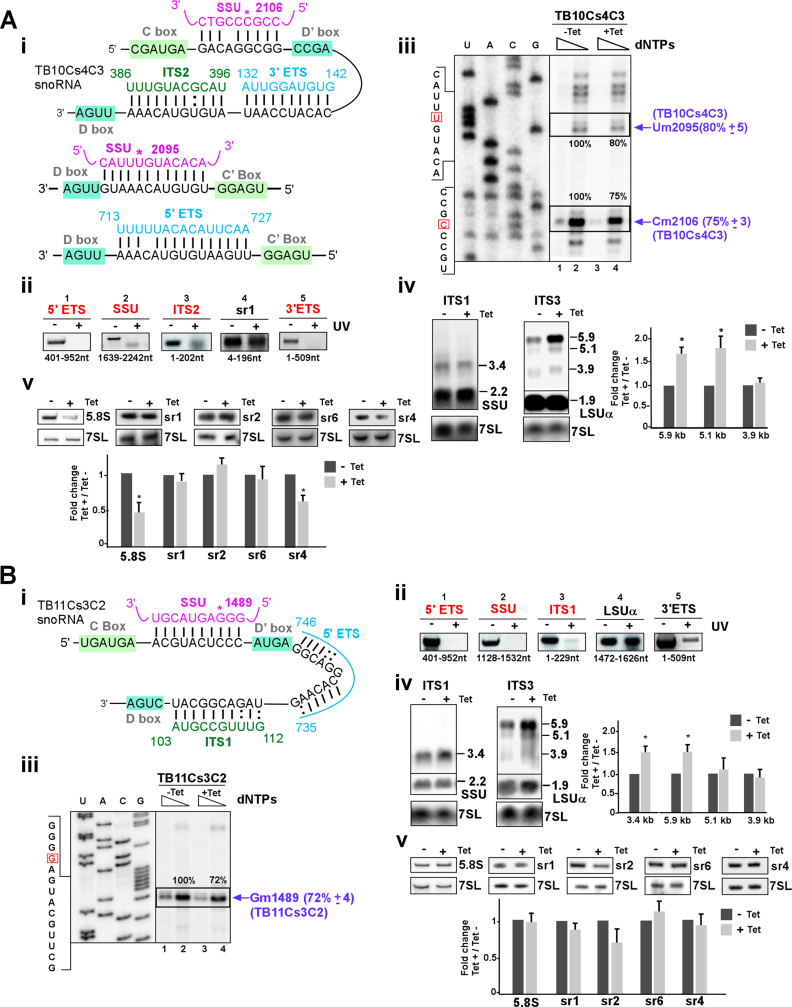
The role of TB10Cs4C3 and TB11Cs3C2 snoRNAs in pre-rRNA processing. (**A**) **TB10Cs4C3 snoRNA** (**i**) The potential for base-pair interaction between TB10Cs4C3 and pre-rRNA with respect to the C and D boxes. (**ii**) ‘RNA walk’ analysis of TB10Cs4C3 snoRNA. RNA was prepared from irradiated cells (+UV) and from control untreated cells (−UV) and subjected to affinity selection using anti-sense biotinylated oligonucleotide complementary to TB10Cs4C3 snoRNA. For other details see the legend to Figure 3A (ii). (**iii**) Validation of reduction in the level of Nms as a result of TB10Cs4C3 snoRNA silencing. Total RNA (8 μg) from TB10Cs4C3 before (−Tet) or after 3 days of silencing (+Tet) was subjected to primer extension (Supplementary table S1) using high- and low-dNTP concentrations (1 and 0.004 mM). For other details, see the legend to Figure 3A (iii). (**iv**) rRNA processing defects after TB10Cs4C3 silencing. The legends are same as for Figure 3A (iv), with total RNA isolated from TB10Cs4C3 silenced cells. (**v**) Reduction in the level of srRNAs following silencing of TB10Cs4C3. For other details, see legends in Figure 3A (v) but total RNA from TB10Cs4C3 silenced cells was used. (**B**) **TB11Cs3C2 snoRNA (i)** The potential base-pair interaction between TB11Cs3C2 and pre-rRNA with respect to the C and D boxes. The predicted Nm site guided by TB11Cs3C2 is marked by an asterisk. (**ii**) ‘RNA walk’ analysis of TB11Cs3C2. Same as in Figure 3A (ii) but anti-sense biotinylated oligonucleotide complementary to TB11Cs3C2 snoRNA was used for the affinity selection. The validated interaction domain between TB11Cs3C2 snoRNA and rRNA site is indicated. (**iii-v**) The legends are same as for Figure 3A (iii-v), but the analysis was performed using RNA isolated from TB11Cs3C2 snoRNAi cell lines that were uninduced (−Tet) or tet-induced (+Tet) for 3 days.

The TB10Cs4C3 snoRNA was distributed between early complexes (fractions 17–23) but also accumulated in later pre-40S complexes (fractions 9–11) (Figure 2A). Bioinformatics approaches predicts overlapping interactions for TB10Cs4C3 snoRNA with distinct rRNAs in both the proximal and distal domains of the pre-LSU rRNA sequence (ITS2 and 3′ ETS) (Figure 4A (i)). Two methylation target sites were also predicted for TB10Cs4C3 on SSU Um2095 which is also pseudouridylated (45), and Cm2106 (Figure 4A (i)). snoRNA base-pairing interaction with 5′ ETS is located directly upstream of D box (Figure 4A (i)). The function of TB10Cs4C3 was investigated to gain further support for the coordination between proximal and distal processing events. Using the ‘RNA walk’ approach, we provide evidence for the interactions of TB10Cs4C3 with 5′ ETS, SSU, ITS2 and 3′ ETS domains along with controls showing the absence of stops on domains not expected to be involved in the interactions (Figure 4A (ii) and Supplementary Figure S3A (i)). Analysis of the chimeric cross-linked RNA duplexes supports the interaction of the snoRNAs with 5′ and 3′ETS. Notably, we failed to detect interaction of the TB10Cs4C3 snoRNA with ITS2 by chimera analysis (Supplementary Figure S3A (ii)).

To determine whether TB10Cs4C3 snoRNA involved in pre-rRNA processing, silencing of the TB10Cs4C3 snoRNA was verified (Supplementary Figure S3A (iii)) and led to the reduction in the predicted Nm modifications at the 3′ end of SSU (Um2095 and Cm2106) (Figure 4A (iii)). Note that stronger RT stop due to Nm at Cm2106 position was observed. Next, we examined the effect of TB10Cs4C3 silencing on rRNA precursor level. Silencing induced clear pre-rRNA defects with accumulation of mainly 5.9 and 5.1 Kb LSU precursors (Figure 4A (iv) and Supplementary Figure S3A (iv)). The effect on small RNAs indicated reduction in the level of both 5.8S rRNA and sr4 (Figure 4A (v)). Interestingly, no effect on SSU processing was observed, suggesting that this RNA has a dual function to guides methylations on SSU and functions in LSU processing. Additionally, the effect on both 5.8S rRNA and sr4 suggested cross-talk between processing events at the 5′ and 3′ ends of LSU precursor.

#### TB11Cs3C2 snoRNA

TB11Cs3C2 snoRNA is found mostly in the smaller RNP complexes (fractions 1–7, ∼20S–30S) but also exhibits a distinct peak on the pre-40S/pre-60S complexes (fractions 9–13) (Figure 2A). The base pairing interactions between TB11Cs3C2 snoRNA and sequences within 5′ETS, SSU and ITS1 are proposed (Figure 4B (i)). The ‘RNA walk’ enabled mapping of the snoRNA TB11Cs3C2 interactions with all three sites within the SSU domain (Figure 4B (ii)). To gain further support for predicted snoRNA–rRNA interaction, cross-linked species were analyzed by chimera ligation method. The data revealed only the interactions of the snoRNAs with the 5′ ETS but TB11Cs3C2–ITS1 interaction was not detected above the background level (Supplementary Figure S3B (ii)). The TB11Cs3C2 snoRNA transcript was then silenced (Supplementary Figure S3B (iii)), and the mapping of the Nm (SSU-Gm1489) showed that modification on the predicted site was significantly reduced when snoRNA silenced (Figure 4B (iii)). Silencing also resulted in the accumulation of the 3.4 Kb pre-SSU as well as the accumulation of the 5.9 Kb pre-LSU precursor (Figure 4B (iv) and Supplementary Figure S3B (iv)). Furthermore, northern blot analysis probing the level of srRNAs showed no significant changes upon TB11Cs3C2 snoRNA silencing (Figure 4B (v)). The accumulation of 3.4 Kb pre-rRNA indicates that efficiency of processing SSU precursor after ITS1 cleavage into mature SSU was reduced in the TB11Cs3C2 silenced cells. The interaction with ITS1 although not supported by the chimera analysis is supported by both the ‘RNA walk’ and the defect in SSU processing under silencing. Therefore, these data suggest that TB11Cs3C2 snoRNA has a dual function in both rRNA processing and modification.

#### TB10Cs1C4 snoRNA

**Figure 5.**
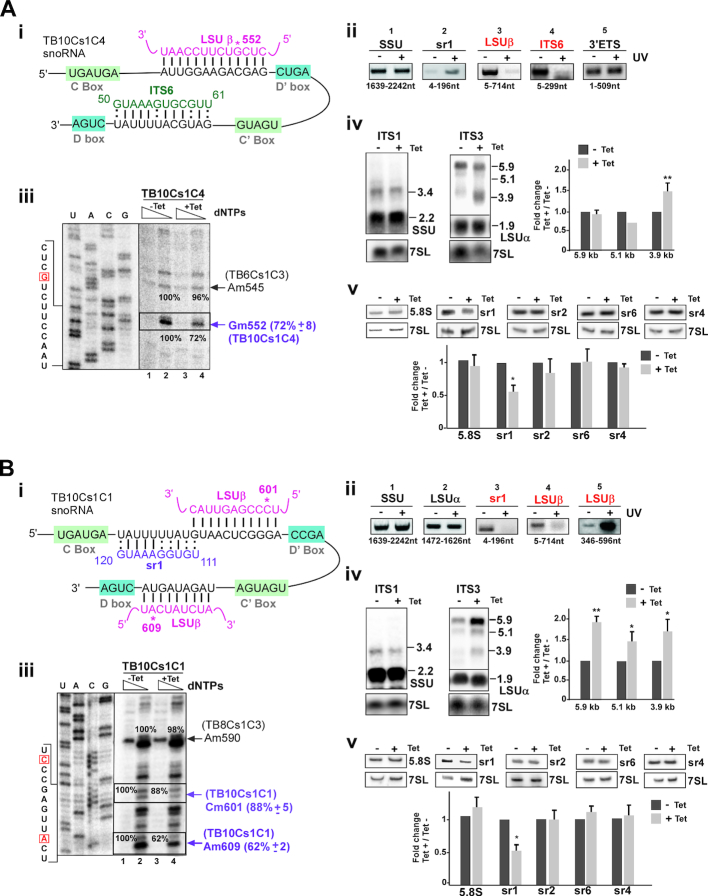
The role of TB10Cs1C4 and TB10Cs1C1 snoRNA in pre-rRNA processing. (**A**) **TB10Cs1C4 snoRNA** (**i**) Predicted TB10Cs1C4 and pre-rRNA interactions with respect to the C and D box motifs indicated. 2′*-O-*methylated nucleotide (Gm552) is marked by an asterisk. (**ii**) ‘RNA walk’ analysis of TB10Cs1C4. The experiment was performed as in Figure 3A (ii), but the RNA was subjected to affinity selection with anti-sense biotinylated oligonucleotide complementary to TB10Cs1C4 snoRNA. (**iii**) Validation of reduction in the level of Nm as a result of TB10Cs1C4 snoRNA silencing. For other details, see the legend to Figure 3A (iii).The mean percentage of band intensity corresponding to Nm stop on the rRNA in the TB10Cs1C4 silenced cells (compare lane 2, −Tet to lane 4, +Tet) with the standard deviation based on two independent experiments is given at the right. (**iv**) Silencing of TB10Cs1C4 leads to accumulation of pre-rRNA precursors. Northern blotting was performed as in Figure 3A (iv) but total RNA was harvested from TB10Cs1C4 snoRNAi cells that were uninduced (−Tet) or tet-induced (+Tet) for 3 days. (**v**) Reduction in the level of srRNAs following silencing of TB10Cs1C4. Northern blot analysis was performed as in Figure 3A (v) but RNA was prepared from TB10Cs1C4 snoRNAi cells that were tet-induced or uninduced for 3 days. (**B**) **TB10Cs1C1 snoRNA** (**i**) The potential base-pair interactions between TB10Cs1C1 and pre-rRNA with respect to C and D box motif. The predicted 2′-*O*- methylation sites for TB10Cs1C1 are marked by an asterisk. (**ii**) ‘RNA walk’ analysis of TB10Cs1C1. The legends are same as described in Figure 3A (ii) but analysis performed for the TB10Cs1C1-precursor rRNA interaction. (**iii-v**) The legends are same as for Figure 3A (iii-v), but the analysis was performed using total RNA isolated from TB10Cs1C1 snoRNAi cell lines that were uninduced (−Tet) or tet-induced (+Tet) for 3 days.

This snoRNA co-sedimented with both large and smaller rRNA processing complexes (Figure 2A). The interaction domain of the snoRNA with LSUβ, where this snoRNA guides a modification at LSUβ-Gm552, and with ITS6 is demonstrated (Figure 5A (i)). These two base pair interactions were verified by ‘RNA walk’ (Figure 5A (ii) and Supplementary Figure S4A (i)). In agreement with ‘RNA walk’, the chimera analysis showed marked enrichment of TB10Cs1C4 snoRNA-ITS6 species, while almost no difference was observed at the remaining ITS regions (Supplementary Figure S4A (ii)).

TB10Cs1C4 snoRNA was silenced and efficient silencing was observed (Supplementary Figure S4A (iii)). Notably, the silencing of the snoRNA resulted in the strong reduction in the level of the proposed guided modification at LSUβ-Gm552 (Figure 5A (iii)). However, no change in adjacent methylation was detected for Am545 (guided by TB6Cs1C3) under TB10Cs1C4 silencing (Figure 5A (iii)). Silencing of the TB10Cs1C4 snoRNA resulted in accumulation of only the 3.9 Kb precursor whereas reduction in level of 5.9 and 5.1 kb pre-rRNA found with ITS3 probe (Figure 5A (iv) and Supplementary Figure S4A (iv)). The results for srRNA probe hybridization showed reduced level of mature sr1 (Figure 5A (v)). Surprisingly, no effect on srRNA-2,4 and 6 was found, despite the interaction with ITS6, which was verified via ‘RNA walk’ and chimera analysis, suggesting that this interaction does not affect rRNA processing. This observation suggest that TB10Cs1C4 is involved in processing of 3.9 Kb pre-rRNA which guides methylation on LSUβ-G552. We can conclude that Nm modification on LSU is critical and the processing defects are an outcome of the loss of this modification (see below). The role of modification in ribosome biogenesis is the subject of ongoing studies.

#### TB10Cs1C1 snoRNA

The TB10Cs1C1 snoRNA was found not only in early complexes (fraction 19–21) but also in late pre-60S ribosomal complexes (fractions 11–15) (Figure 2A). The proposed interaction domains between snoRNA and target RNA are presented (Figure 5B (i)). TB10Cs1C1 snoRNA has the potential to base-pair with sr1, and the interaction was verified by ‘RNA walk’ (Figure 5B (ii) and Supplementary Figure S4B (i)). Specific enrichment of TB10Cs1C1 snoRNA-ITS3 pre-rRNA chimera was detected (Supplementary Figure S4B (ii). To demonstrate whether TB10Cs1C1 is required for site-specific methylation of LSUβ at two predicted sites (LSUβ−Cm601 and LSUβ−Cm609), the snoRNAs was silenced (Supplementary Figure S4B (iii) and Nm modifications were mapped using the low dNTP method on two guided sites. TB10Cs1C1 snoRNA silencing showed a noticeable but minor reduction in Nm at Cm601 site, but very strong reduction observed at Cm609 site (Figure 5B (iii)). Silencing of TB10Cs1C1 snoRNA resulted in the accumulation of all LSU precursors (5.9, 5.1, 3.9 Kb pre-rRNA). Mature sr1 level was reduced after snoRNAi induction (Figure 5B (iv and v) and Supplementary Figure S4B (iv)). Taken together, our results suggest that TB10Cs1C1 function in guiding modification on LSUβ and in processing of LSU. In summary, these data demonstrate that multiple snoRNAs implicated in processing of pre-rRNA have been psoralen crosslinked to pre-rRNA and associated with larger processing complexes which possibly contain pre-rRNA.

#### snoRNAs having no predicted interaction domains with intervening sequences but affect rRNA processing

Sedimentation shown in Figure 2A show that some of the *T. brucei* snoRNAs (TB8Cs1C1, TB9Cs2C3, TB9Cs3H2) are found mainly in a single population in the smaller complexes as a monoparticle cosedimenting with the ∼20S–30S. These snoRNPs have been predicted to guide modification on rRNA but does not base-pair with the precursor rRNA. We therefore examined the possibility that whether these additional snoRNAs may also be involved in rRNA processing.

#### TB8Cs1C1 snoRNA

**Figure 6.**
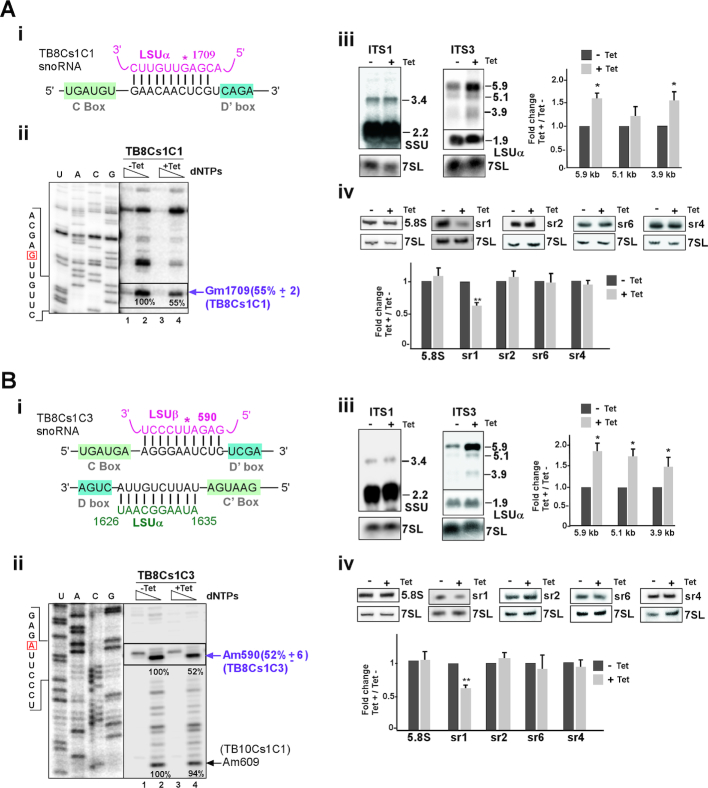
The role of TB8Cs1C1 and TB8Cs1C3 snoRNA in pre-rRNA processing. (**A**) **TB8Cs1C1 snoRNA** (**i**) The potential base-pairing interaction between TB8Cs1C1 snoRNA and rRNA with respect to the C and D’ boxes. The predicted 2′-*O*- methylation site for TB8Cs1C1 is marked by an asterisk. (**ii**) Validation of reduction in the level of Nm as a result of snoRNA silencing. The legends are same as described in Figure 3A (iii), but the analysis was performed using total RNA isolated from TB8Cs1C1 snoRNAi cell lines that were uninduced (−Tet) or tet-induced (+Tet) for 3 days.The mean percentage of band intensity corresponding to Nm stop on the rRNA in the TB8Cs1C1 silenced cells (compare lane 2, −Tet to lane 4, +Tet) with the standard deviation based on two independent experiments is given at the right. (**iii**) Northern blot analysis of rRNA precursors following the silencing of TB8Cs1C1 snoRNA. Total RNA was prepared from cells carrying the TB8Cs1C1 snoRNAi silencing construct without induction (−Tet) or after tetracycline induction for 3 days (+Tet). pre-rRNA species detected by hybridization with a probe to ITS1 and ITS3 region. statistical analysis was performed same as described in Figure 3A (iv). (**iv**) Reduction in sr1 level following silencing of TB8Cs1C1. Total RNA (5 μg) was separated on a 10% denaturing gel and subjected to northern analysis with the different srRNA probes. Statistical analysis was performed as in Figure 3A (iv). (**B**) **TB8Cs1C3 snoRNA** (i) The potential base-pairing interaction between TB8Cs1C3 snoRNA and rRNA with respect to the C and D’ boxes. The predicted 2′-O- methylation site is marked by an asterisk. (**ii)**The legends are same as described in Figure 3A (iii), but the analysis was performed using total RNA isolated from TB8Cs1C3 snoRNAi cell lines that were uninduced (−Tet) or tet-induced (+Tet) for 3 days. (**iii-iv**) same as in panel A(iii-iv) but for the TB8Cs1C3 snoRNA.

Density gradient sedimentation analyses demonstrated that TB8Cs1C1 snoRNA is associated with late pre-60S complexes (fraction 13–15, Figure 2A) and has no proposed interaction domains in ITS/ETS regions. TB8Cs1C1 snoRNA utilizes box D’ for guiding methylation on LSUα rRNA (Gm1709) (Figure 6A (i)). To investigate its role in rRNA processing, the snoRNA was silenced and its depletion was verified by northern analysis (Supplementary Figure S5A (i)). Silencing of TB8Cs1C1 snoRNA resulted in significant reduction of LSUα-Gm1709 (Figure 6A (ii)).

The pattern of reduced methylation in snoRNA silenced cells indicated that the rRNA processing events that produce precursors appear much larger amount than usual amount. To confirm this, northern blot analysis was performed. The result of ITS3 probe revealed a major accumulation of both 5.9 and 3.9 Kb LSU precursors when TB8Cs1C1 snoRNA silenced (Figure 6A (iii) and Supplementary Figure S5B (i)). Furthermore, reduction in the level of srRNA1 was confirmed (Figure 6A (iv)). Indeed, the rRNA processing defects observed as a result of snoRNA silencing may suggest that either the folding of rRNA governed by this snoRNA affects pre-rRNA processing, or that processing is influenced by the lack of these specific methylation modifications. It is possible that the effect on rRNA folding slows down the normal processing events resulted in accumulation of the pre-rRNA. However, the defect in sr1 processing indicates a specific effect on rRNA processing.

#### TB8Cs1C3 snoRNA

Sedimentation profile of TB8Cs1C3 appeared very similar to those of TB8Cs1C1, detected with free snoRNPs and, interestingly, co-migrated with late, pre-60S complexes (Figure 2A). The interaction domain between the TB8Cs1C3 snoRNA and its target on LSUβ is presented (Figure 6B (i)). snoRNA depletion was examined by northern blotting (Supplementary Figure S5A (ii)). We checked for a rRNA primer extension pause at guided Nm site on LSUβ-Am590 under TB8Cs1C3 silencing and found strong reduction in 2′-*O*-methylation as compared to uninduced cells (Figure 6B (ii)). Notable, no significant reduction in level of neighboring Nm guided by another snoRNA was observed (Figure 6B (ii)).

Silencing of the snoRNA, also showed significant accumulation of all LSU rRNA precursors (5.9, 5.1 and 3.9 Kb pre-rRNA) (Figure 6B (iii) and Supplementary Figure S5B (ii)). In addition, there was a marked decrease in the level of mature sr1 (Figure 6B (iv)). Taken together, these results suggest that TB8Cs1C3 functions in both guiding methylation and processing of an LSU precursor.

#### TB9Cs2C3 snoRNA

**Figure 7.**
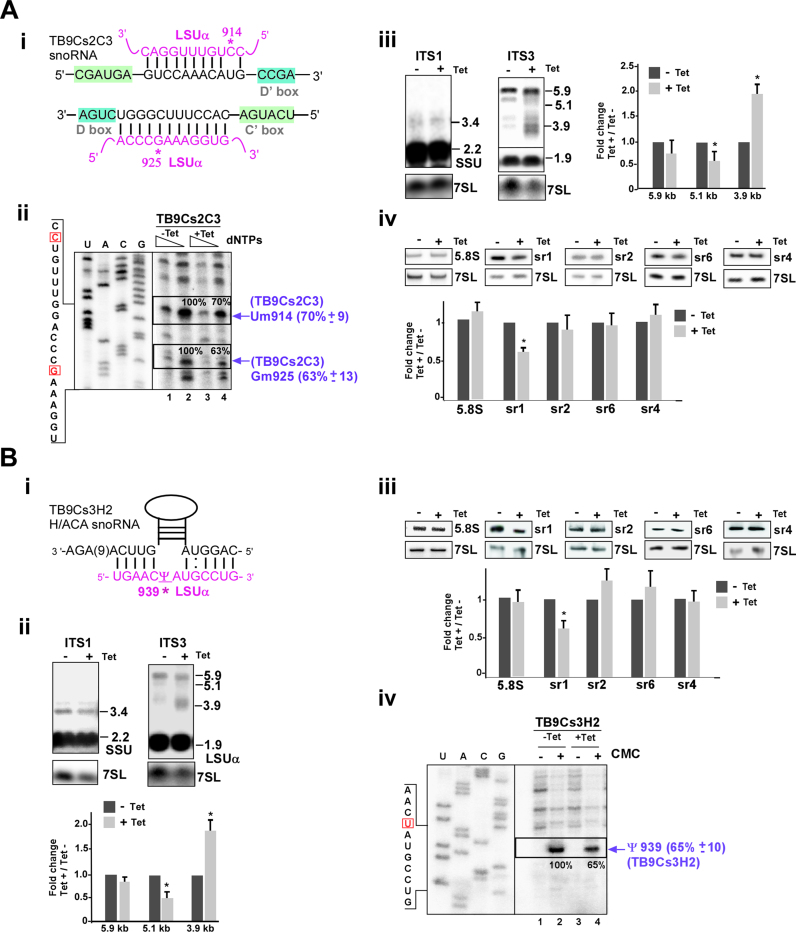
The role of TB9Cs2C3 and TB9Cs3H2 snoRNA in pre-rRNA processing. (**A**) **TB9Cs2C3 snoRNA** (**i**) The potential base-pair interaction between TB9Cs2C3 and rRNA with respect to the C and D boxes. 2′*-O-*methylated nucleotides of LSUα (Um914 and Gm925) are indicated. (**ii-iv**) The legends are same as described for Figure 6A (ii-iv), but the analysis was performed using RNA isolated from TB9Cs2C3 snoRNAi cell lines that were uninduced (−Tet) or tet-induced (+Tet) for 3 days. (**B**) **TB9Cs3H2 snoRNA** (**i**) The potential base-pair interaction between TB9Cs3H2 H/ACA snoRNA and LSUα rRNA in the pseudouridylation pocket. The position of the pseudouridylated nucleotide is marked with an asterisk. (**ii**) rRNA processing defect in TB9Cs3H2 silenced cells. Same as in Figure 3A (iv) but for the RNA isolated from TB9Cs3H2 snoRNAi cell lines that were uninduced (−Tet) or tet-induced (+Tet) for 3 days. (**iii**) Reduction in the level of srRNAs following silencing of TB9Cs3H2. Total RNA (5 μg) was separated on a 10% denaturing gel and subjected to northern analysis with the different srRNA probes. Statistical analysis was performed as described for Figure 3A(iv). (**iv**) Experimental confirmation of Pseudouridylation (Ψ) in LSUα by CMC-primer extension for TB9Cs3H2 snoRNA. Total RNA (15 μg) was prepared from procyclic *T. brucei* cells carrying TB9Cs3H2 without induction (−Tet) or after 3 days of induction (+Tet). The treated RNA (+CMC) and control RNA (−CMC) were subjected to primer extension with primers shown in Supplementary Table S1. Extended products were separated and analyzed on an 8% polyacrylamide –7 M urea gel along with a DNA sequencing ladder produced using the same primer. Partial DNA sequences are given. Locations of the pseudouridine (Ψ) guided by TB9Cs3H2 on LSUα rRNA is indicated by arrow. The mean percentage of band intensity corresponding to Ψ signal on the rRNA in the TB9Cs3H2 silenced cells (compare lane 2, to lane 4,) with the standard deviation based on two independent experiments is given at the right.

TB9Cs2C3 snoRNA contain two guide domains that target methylation at more than one site on LSUα (Um914 and Gm925) (Figure 7A (i)). This snoRNA mostly co-sedimented with late pre-60S processing complexes (Figure 2A) and silencing of the TB9Cs2C3 snoRNA (Supplementary Figure S5A (iii)) resulted in significant reductions in the level of the Nm modifications at both nucleotides (Um914 and Gm925) (Figure 7A (ii)). snoRNA silencing also resulted in increase in the level of only 3.9 Kb LSU precursor (Figure 7A (iii) and Supplementary Figure S5B (iii)) and the level of sr1 was reduced significantly (Figure 7A (iv)).

All together, these data demonstrate that TB9Cs2C3 snoRNA silencing has a severe impact on both pre-LSU rRNA processing and methylation.

#### TB9Cs3H2 snoRNA

It is not much known about H/ACA snoRNA function in rRNA processing. In yeast, snR10 and snR30 participate in rRNA processing (10). TB9Cs3H2 is the only *T. brucei* H/ACA snoRNA examined in the current study and guides Ψ on LSUα-939 (Figure 7B (i)) (45). TB9Cs3H2 snoRNA was detected with free snoRNPs and co-migrated with large early processing complexes (fraction 17–19 in Figure 2A). To study the effect of TB9Cs3H2 snoRNA silencing in pre-rRNA processing pathway, RNA after TB9Cs3H2 snoRNAi was examined (Supplementary Figure S5A (iv)). Figure 7B (ii) shows the result of northern hybridization of RNA extracted from TB9Cs3H2 snoRNA silenced cells. 3.9 Kb rRNA precursor accumulated more upon silencing whereas 5.9 and 5.1 kb pre-rRNA detected with ITS7 probe did not differ significantly between un-induced and induced snoRNAi cells (Supplementary Figure S5B (iv)). Mature sr1 level seen reduced further confirmed the inhibition of processing at ITS3 region (Figure 7B (iii)).

It is intriguing that an rRNA processing defect was observed as result of silencing of a H/ACA snoRNA, which mostly guide modifications. The effect of silencing on the level of the Ψ was examined by the CMC method. CMC remains attached only to pseudouridine following alkaline hydrolysis, and its presence results in a strong stop, 1 nt before the modified nucleotide in a primer extension assay (69). The result showed significant reduction in the level of the guided Ψ as a result of silencing (Figure 7B (iv)). Thus, Ψ modification at this site is essential for proper rRNA processing.

All together these finding establish dual function for *T. brucei* snoRNPs which not only part of methylating complexes but also sediments with processing complexes through base pairing to flanking precursor sequences or rRNA.

#### snoRNAs affecting the liberation of sr1 guide modifications in the ribosome spanning the protein exit tunnel to the sr1 position

**Figure 8.**
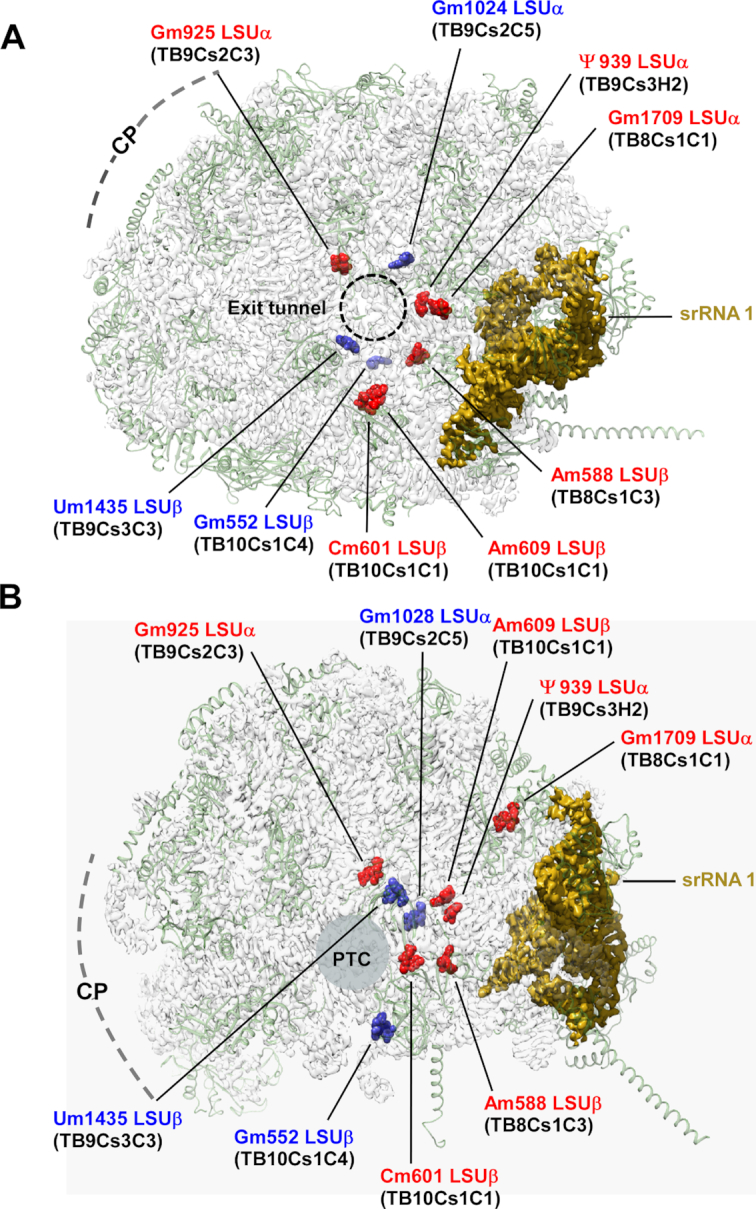
snoRNAs shown to affect srRNA1 processing guide rRNA modifications at a ribosomal region that spans the protein exit tunnel to the site of srRNA1 localization. 3D localization of rRNA modifications guided by snoRNAs whose depletion altered srRNA1 liberation. (**A**) Top view from the surface of a solvent-exposed ribosome indicating the proximity of seven such rRNA modifications to the protein exit tunnel. The protein exit tunnel is indicated by a dashed circle, central protuberance (CP) localization is highlighted with a dashed arc. (**B**) Side view demonstrating the proximity of the modified residues to the srRNA1 location in the mature ribosome. rRNA is presented as a solid surface, ribosomal proteins are indicated as cartoons. srRNA1 is colored dark yellow. rRNA modifications are shown in red to indicate positions of modifications mediated by snoRNAs that operate only on the mature ribosome, and blue for residues whose snoRNA also interacts with intergenic regions. CP and PTC are marked for orientation. snoRNA numbering as well as residue numbers in the mature *Trypanosoma brucei* ribosome are indicated in the figure.

One of the most peculiar findings of our study suggested that knockdown of certain snoRNAs, which guide modifications on the LSU or do not interact with ITSs, induced major rRNA processing defects that alter the liberation of sr1. These findings suggested a functional role of snoRNAs that is distinct from direct involvement in cleavage events via interactions with intervening sequences. In an attempt to further understand these observations, we explored the localization of these modified positions in the recently reported 3D atomic resolution structures of trypanosome ribosomes (52,54,74). As anticipated, these snoRNA species, which are highly conserved throughout trypanosomes (75), were indeed shown to mediate rRNA modifications in both *Trypanosoma* and *Leishmania* species, and the modified residues were clearly visualized in the high resolution map densities of mature ribosome samples derived from *T. cruzi* epimastigotes (52) and *L. donovani* promastigoes (54,74). Surprisingly, the localization of the modified residues was restricted to a ribosomal region that spans between the peptide exit tunnel and the srRNA1 site in the mature ribosome, with most modifications closely surrounding the protein exit tunnel but also near PTC (Figure 8A and B). Exit tunnel formation was similarly shown to occur during late yeast ribosome biogenesis stages (55) and these stages were also recently linked with the folding of the sr1 homologous region in yeast (26). The observation that alteration of a snoRNA guiding rRNA modifications within a ribosomal region suggested to fold along with srRNA1 during ribosome biogenesis results in sr1 processing defects. These observations strongly imply that rRNA modifications might serve as check-points that condition the liberation of sr1 on proper rRNA folding. Given the high abundance of snoRNAs mediating these modifications in trypanosomes, we also suggest that these modifications serve as critical check-points in trypanosomatid ribosome biogenesis, and that their high abundance is necessary to ensure that those critical positions will be modified on every ribosome.

## DISCUSSION

The processing of trypanosome rRNA is unique compared to other eukaryotes due to the additional cleavage events needed for the generation of the two long and four short LSU rRNA segments. In this study, we provide functional evidence for the involvement of ten snoRNAs in trypanosomatid-specific rRNA processing events. We find that these snoRNAs can be divided into two families, those that participate in early or late LSU processing events. Five of the snoRNAs whose depletion resulted in defective srRNA1 processing were shown to guide six modifications spanning between the protein exit tunnel and sr1 in the ribosome, suggesting that these modifications may serve as check-points for the final step of rRNA processing, which give rise to the liberation of sr1. Moreover, our data suggest that processing of *T. brucei* LSU is post-transcriptional, and that the first small rRNA which is transcribed is the last to be processed.

### The machinery that guides the trypanosome-specific processing

It was not known for a long time how this extra trypanosome LSU rRNA processing is mediated, and whether snoRNAs are involved in this process. The need for unique snoRNAs to carry out these processing steps was suggested by our group when inspecting the rRNA precursors that accumulated during *Nop1* (C/D snoRNA pathway) and *Cbf5* (H/ACA pathway) depletion (41,42). Both previous studies (43,44) and the current one were designed to assign specific snoRNAs to particular trypanosome-specific processing needs.

Interestingly, this study highlighted several snoRNAs that are implicated in srRNA1 liberation; these could be divided into two families, snoRNAs that directly interact with ITS3 and ITS4, which flank srRNA1 or srRNA1 itself (TB9Cs2C5, TB9Cs3C3, TB10Cs1C1) and those that guide modifications on mature rRNA segments and do not interact directly with srRNA1 (TB8Cs1C1, TB8Cs1C3, TB9Cs2C3, TB9Cs3H2). Altogether, our data indicate that liberation of srRNA1 is the most tightly controlled processing event identified in this study. It is possible that different snoRNAs could assist in cleavage reaction at same ITS3 site.

**Figure 9.**
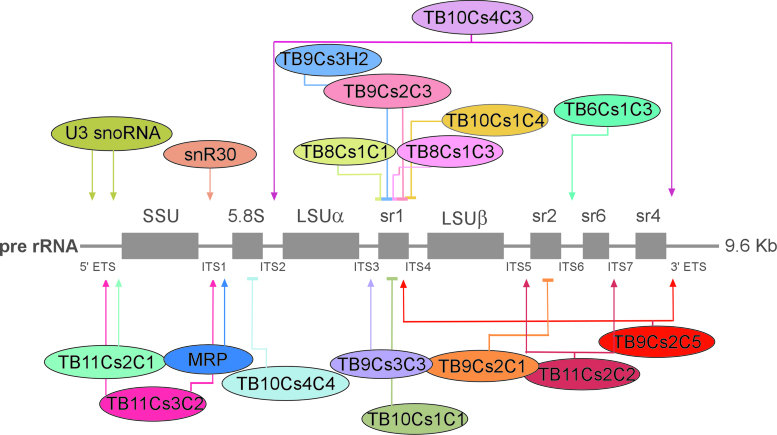
**snoRNAs involved in rRNA processing in *Trypanosoma brucei***. Schematic representation of rRNA domains affected by depletion of *Trypanosoma brucei* snoRNAs. Closed arrows designate snoRNAs that direct interact with ITS and/or ETS sequences and directly involved in rRNA processing events. Inhibitory sign mark snoRNAs affecting the processing of srRNAs due to guiding essential modification mostly in the ribosome exit tunnel.The domains affected are indicated based on our previous studies (42–44) and the current study.

Based on our current results and our previous studies (43,44), release of other srRNAs is also orchestrated by snoRNAs (Figure 9). RNA walk and Chimera ligation method provided support for the proposed interactions. Our previous studies indicated that cleavage at ITS5 and ITS6 is most likely controlled by snoRNA TB9Cs2C1 and TB11Cs2C2 (43,44). Only a single very abundant snoRNA is implicated in the processing of ITS7 (TB11Cs2C2), and processing at 3′ ETS is most probably assisted by TB9Cs2C5 and TB10Cs4C3 snoRNA described in this study (Figure 9). In yeast, this step is mediated very early in the processing cycle by Rnt1p (76). The current study also revealed two snoRNAs that suggest the existence of cross-talk and coordination of early stages of processing between the 5′ and 3′ end of the 5.9 Kb LSU pre-rRNA. TB9Cs2C5 can interact simultaneously with srRNA1 and 3′ ETS, and in addition, can affect the liberation of srRNA1 and srRNA4. Indeed, such interactions during processing were suggested by Liu *et al.* (52). Our data indicate that TB9Cs3C3 snoRNA binds in ITS3-sr1 region suggesting that snoRNA could direct processing machinery where cleavage occurs. Most interesting is the function of TB10Cs4C3, which interacts with ITS2 and 3′ETS and affects the liberation of 5.8S and sr4, further supporting the cross-talk between processing events taking place at two distal sites on the LSU.

Surprisingly, in most of the snoRNA silenced cell lines, the accumulation of the 5.9 Kb LSU precursor was not accompanied by reduction in the level of most of the small rRNA species other than srRNA1. Using Trypanosome cell permeable system, we showed that silencing of snoRNA resulted in enhanced polymerase I nascent rRNA transcription (compare lanes 4–6 to lanes 1–3, Supplementary Figure S6). Thus, the accumulation of 5.9 Kb pre-rRNA observed in cells silenced for snoRNA likely reflects enhancement in transcription of rRNA gene clusters compensating for the defects in rRNA biogenesis. Nevertheless, we cannot rule out the possibility that lack of snoRNP machinery cause slowdown in the rRNA processing thus resulting in precursor accumulation. Such a direct or indirect effect could influence the 5.9 Kb pre-rRNA processing.

Taken together, with these observations, supports the notion that the cleavage of srRNA2, -4 and -6 takes place before the cleavage of srRNA1. These findings correlate well with the recent observations from the Cryo-EM studies of *L. donovani* ribosomes, suggesting that the assembly of srRNA2, -4 and -6 occur during early ribosome maturation steps, whereas the processing of srRNA1 is the last step to occur (54). Our studies are also consistent with the recent examinations of *T. cruzi* ribosome structures, which suggested cross-talk between srRNA1 and srRNA4 processing during late biogenesis steps (52). Studies in yeast suggested that the r-proteins are incorporated into the ribosome in a hierarchical manner during early, middle or late biogenesis steps. These assembly steps begin at the solvent exposed side, followed by exit tunnel formation, and are completed by the formation of inter-subunit interface and the central protuberance (55,77). The recently reported Cryo-EM structures of *T. cruzi* and *L. donovani* ribosomes suggest a similar dogma in trypanosome ribosome biogenesis, whereby srRNA-2, -4 and -6 are assembled, and potentially processed, at early biogenesis steps, and srRNA1 is assembled at later stages (52,54). These studies also linked the order of assembly with the incorporation of r-proteins in *T. cruzi* ribosomes, where srRNA2 (sr2) is anchored by uL3 (previously L3), and srRNA6 (sr6) anchored by eL6 (L6) and eL33 (L35A), all early-acting proteins (52), whereas srRNA1 and srRNA4 were shown to associate with the middle-acting proteins eL19 (L19), eL34 (L34) and eL31 (L31) during ribosome assembly (52). Similar observations were reported for *L. donovani* ribosomes (54).

### sr1 liberation is the final step of LSU processing, which is controlled by snoRNAs guiding methylation along the protein exit tunnel

It was not clear how snoRNAs, which guide the modifications on the LSU, affect the processing of sr1. However, mapping the localization of the modified positions to the recently reported 3D structures of mature ribosomes, indicated that most modifications guided by snoRNAs, which also affect sr1 liberation, are localized around the protein exit tunnel (Figure 8A) while stretching toward sr1 location in the mature ribosome (Figure 8B). Interestingly, the protein exit tunnel was suggested to fold during late stages of ribosome biogenesis (55), where the folding of the sr1 homolog in yeast was also suggested to occur (26). These data agree with our results suggesting the maturation of srRNA1 during the very same biogenesis stage. The ribosome exit tunnel was proposed to serve as a check-point in ribosome biogenesis, as it was shown to be occupied by the C-terminal extension of the GTPase NOG1 in late pre-60S particles (24). In this study, we show for the first time that methylation along with protein exit tunnel affects rRNA processing, and specifically hinders the last step of processing, which is the liberation of srRNA1. The absence of methylation may affect the binding of r-proteins and impair proper folding of the rRNA. We suggest that rRNA modifications localized to this region may serve as check point to ensure proper local rRNA folding prior to the normal processing of sr1.

### Dual functionality and the variable abundance of snoRNAs

This study, as well as our previous studies (43,44), suggest that in trypanosomes, many of the snoRNAs perform more than a single function; i.e. they both assist in rRNA processing, and guide modifications. Here, we identified several such snoRNAs, including TB9Cs2C5. This snoRNA interacts with 3′ ETS region and guides Nm on LSUα. These activities involve overlapping interaction domains, and thus must be mutually exclusive. In addition, the snoRNA TB10Cs4C3 interacts with ITS2 and affects LSU processing, but also have two modification guide domains in the SSU rRNA; these activities are also mutually exclusive. Similarly, snoRNA TB9Cs3C3 interacts with the ITS regions and guides rRNA modification. In contrast to mammalian snoRNAs, many of the trypanosome guide RNAs can guide more than a single modification in the same or different RNA (45,75,78). Three of the snoRNAs (TB10Cs4C3, TB10Cs1C1, TB9Cs2C3,) examined here contain two guide domains that target 2′-*O*-methylations at more than one site, either in the same or different rRNAs. These guide regions may act exclusively independent; it could be possible that methylations are linked in some fashion and that such coordination perhaps important feature in rRNA biogenesis. The sharp difference in abundance of various trypanosome snoRNAs observed in this and in previous studies (44,45,75) suggested that abundant snoRNAs have special functions. These snoRNAs either assist in rRNA processing, and/or guide modifications on essential rRNA positions. We previously demonstrated a strong correlation between the level of snoRNAs and the modification they guide (45). Notably, among the abundant snoRNAs studied here, two snoRNAs were found to guide modifications near eL41 (L41). These snoRNAs are TB11Cs3C2 and TB10Cs4C3 (Supplementary Figure S7) that guide additional modifications along the protein exit tunnel or are alternatively involved in rRNA processing. eL41 is a eukaryote-specific bridge known to be crucial for ribosome decoding as well as for late ribosome assembly stages (79).

Recent studies using global mapping of 2′-*O-*methylation by RiboMethSeq provided evidence for plasticity in the level of Nm modifications (80,81). In another study, the dynamics of individual 2′-*O-*methylation on rRNA was described using high-throughput 2OMe-seq based on the use of limiting dNTP concentrations (82). Thus, the ribosomes in the cell are a heterogeneous population and not all positions are uniformly modified, except for crucial modifications. However, the 2′-*O*-methylations affect the ability of the ribosome to translate mRNAs (80). In Trypanosomes, the level of modification is developmentally regulated, and this is mediated by controlling the level of the snoRNAs (45). Our study highlights the functional importance of the abundant snoRNAs in guiding domains that are essential for ribosome function. However, this type of regulation is not common to all eukaryotes, since in humans, in which several snoRNAs are known to guide a single modification, no correlation was found between the level of snoRNA and the modification it guides (83).

### Why are so many snoRNAs involved in trypanosome rRNA processing?

snoRNAs involved in rRNA processing function to bring distant sequences into proximity and coordinate the cleavage. Indeed, as mentioned above, this study suggests that the multiple snoRNPs can coordinate the cleavages at both ends of the LSU pre-rRNA. However, snoRNA can also assist in recruiting protein factors to the heart of the processing reactions. It is tempting to speculate that several trypanosome-specific snoRNAs may include protein factor(s) implicated in directing cleavages at specific and unique sites. In addition, LSU processing factors may be replaced by trypanosome-specific snoRNP proteins. The Dbp6p and its associated factors, which was shown to function in releasing the 5.8S rRNA in yeast (84), are missing in the trypanosome genome (50). The function of such a complex could be replaced by a snoRNA such as TB10Cs4C4. The trypanosome genome lacks additional genes encoding factors involved in ITS1 cleavage, such as Rrp17 (50), which was shown to be involved in A3 cleavage in ITS1 (77) and might be replaced by a trypanosome-specific snoRNP protein. It will therefore be of great interest to characterize the protein constituents of each of the snoRNPs involved in processing, and to determine which proteins they contain, and if these proteins are related to the factors that are absent in trypanosomes.

### Why is there such a complex rRNA processing pathway in trypanosomes?

The study raises the question why such an elaborate processing pathway exists in trypanosomes. It was suggested that rRNA fragments are excised to shorten the rRNA and to compensate for the long expansion segments, which are longer than in other eukaryotes (51). However, the question remains why trypanosome rRNA possesses longer expansion segments, including trypanosome-specific ones. ES6S and ES7S are the most significantly enlarged as compared with other eukaryotic ribosomes. Their proximity to the exit tunnel suggests a possible role in translation initiation (52). Of note is ES6S, which has been implicated in the recruitment of the eukaryotic initiation factor 3 (52). Since translational regulation appears to be a prominent regulatory mechanism of gene expression in trypanosomes, binding of additional proteins and translation factors to the ribosomes is required, and such expansion segments may assist in this complex regulation (85). Thus, the specific needs for translational regulation as mentioned above may have led to longer expansion segments. Additional rRNA processing events may have developed to remove parts of the rRNA to shorten the ‘expanded’ rRNA. This imposed new requirements for processing, leading to the appearance of expansion segments, which assist in both the processing and the stabilization of the fragmented LSU.

In summary, this study identifies key events in trypanosome rRNA processing controlled by snoRNAs and highlights the importance of snoRNAs guiding 2′-*O*-methylation along the protein exit tunnel near the PTC as a check-point for ribosome biogenesis. The unique properties of the rRNA processing mechanism and machinery could be harnessed for designing future drugs to inhibit the ribosome function in trypanosomes, in ongoing attempts to fight the devastating diseases caused by these parasites.

## DATA AVAILABILITY

The RNA-seq data reported in this article has been deposited in the Sequence Read Archive (SRA) database (accession number: SRP133196).

## Supplementary Material

Supplementary Data

## Notes

**Disclaimer:** S.M. holds the David and Inez Myers Chair in RNA silencing of diseases.
