# Supplementary material for: Small nucleolar RNAs controlling rRNA processing in Trypanosoma brucei
Source: Nucleic Acids Res. 2019 Jan 3;47(5):2609–29. doi: 10.1093/nar/gky1287 (PMC6411936; doi:10.1093/nar/gky1287)

Supplementary Figure S4

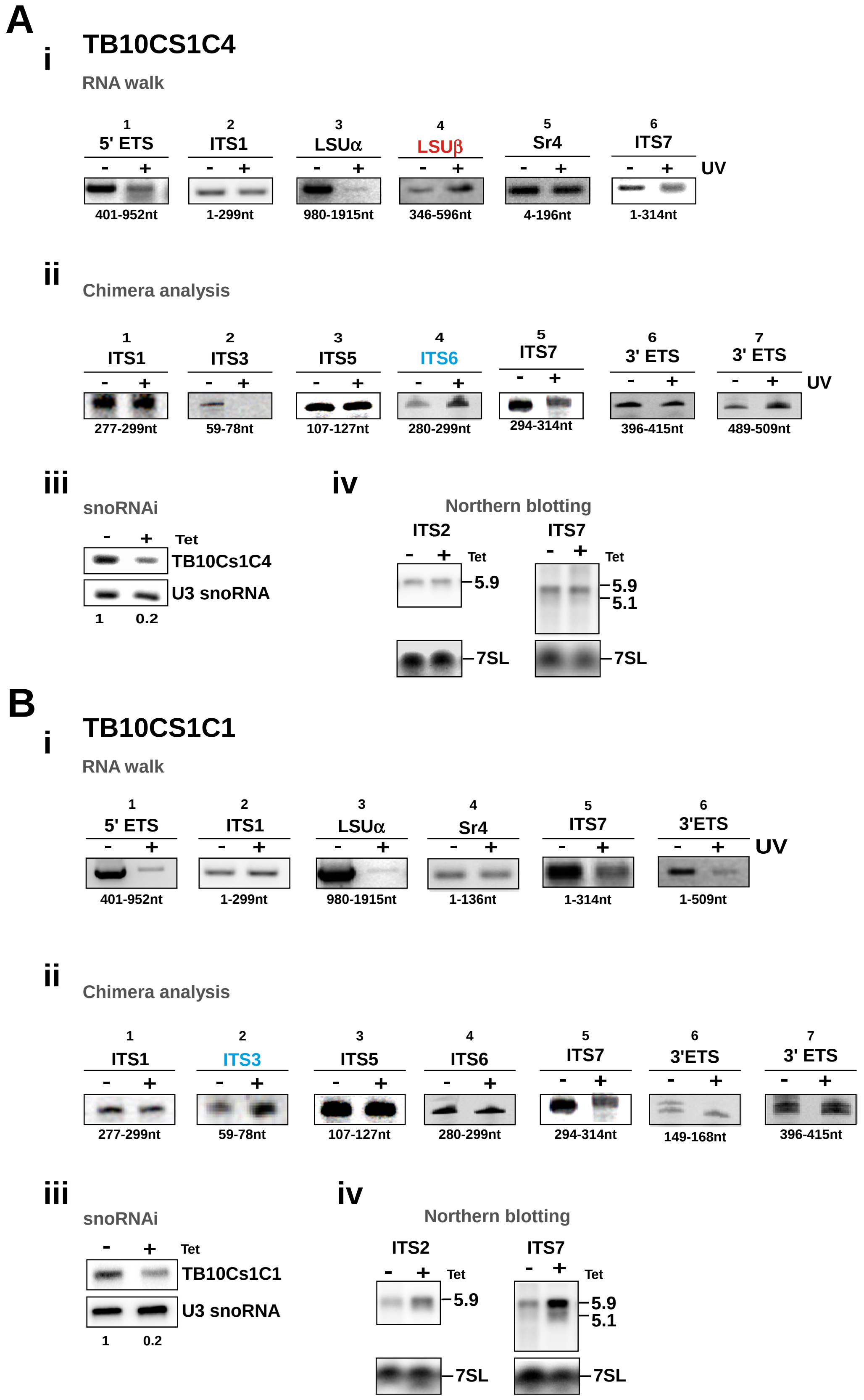

Supplement: Supplementary Data [file gky1287_supplemental_files.zip › Chikne et al Supplementary Figure S4.pdf]
