# Supplementary material for: Small nucleolar RNAs controlling rRNA processing in Trypanosoma brucei
Source: Nucleic Acids Res. 2019 Jan 3;47(5):2609–29. doi: 10.1093/nar/gky1287 (PMC6411936; doi:10.1093/nar/gky1287)
Supplement: Supplementary Data [file gky1287_supplemental_files.zip › Chikne et al_MS_NAR 00948-A-2017.R3 Supplementary Materials.pdf]

Supplementary Materials

Supplementary Table S1. List of primers.

Supplementary Table S2. The RPKM and percentile rank of the snoRNAs in FPLC sizing column fraction and procyclic PRS RNA-seq libraries. The RPKM and the percentile rank are given for snoRNAs in the FPLC sizing column fraction (1) and two biologically-independent procyclic PRS RNA-seq libraries. The designation of the libraries is indicated in the top row. The abundant snoRNAs are highlighted in light green for the FPLC fraction library. Six snoRNAs involved in *T. brucei* pre-rRNA processing events shown (1–3) . The type of snoRNA classes is indicated.

Supplementary Figure S1

SSF1 and TIF6 are localized in the nucleus. **(A).** Procyclic *T. brucei* cells stably expressing PTP (for ProtC-TEV-ProtA)-tagged SSF1 proteins, were fixed with 4% formaldehyde for 20 min followed by immunofluorescence staining with anti-IgG (Sigma) antibodies as described in the Materials and Methods, and detected by a Alexa-Fluro-488- conjugated secondary antibody. Scale bar: 2 µm. **(B).** Procyclic *T. brucei* cells stably expressing eYFP (enhanced yellow fluorescent protein)-tagged TIF6 protein were subjected to live imaging. Scale bar: 10 µm

Supplementary Figure S2

(A) (i) “RNA walk” to identify interacting RNA. Cells were treated with AMT and subjected to UV irradiation; RNA was then subjected to affinity selection with anti-sense biotinylated oligonucleotide complementary to TB9Cs2C5 snoRNA. cDNA was prepared from the affinity-selected RNA, and amplified by PCR, using primers covering the entire rRNA target. The PCR products were separated on a 1% agarose gel and stained with ethidium bromide. RNA from irradiated versus untreated cells is designated by (+) and (-),

respectively. The domains and the amplified fragments are indicated. The validated interaction domains are indicated in red. Domains which are not involved in true base-pairing interactions are indicated in black. **(ii) Validation of U3 C/D snoRNA -5' ETS interactions using chimera analysis.** *Upper panel*; RNA was generated as described in Materials and Methods. RNA from control DNA (-UV) and from the cross-linking experiment (+UV) was subjected to RT-PCR analysis with specific primers (Supplementary table S1) directed to both RNA molecules (U3 snoRNA and 5' ETS pre-rRNA region, indicated). The PCR products were analyzed on 10% polyacrylamide gels. The interaction domain with snoRNAs is indicated. The predicted chimeric interactions are indicated in blue.

Lower panel; **Identification of C/D TB9Cs2C5 snoRNA - precursor rRNA chimeric species.** Chimeric cross linked RNA was generated as described in Materials and Methods. RNA from control (-UV) and following cross linking (+UV) was subjected to RT-PCR analysis with specific primers (Supplementary table S1) directed against both RNA molecules (TB9Cs2C5 snoRNA and pre-rRNA region indicated). The PCR products were analyzed on 10% polyacrylamide gels. The interaction domain with snoRNAs is indicated. The validated snoRNA-target RNA interaction domains are indicated in blue. **(iii) Silencing of TB9Cs2C5 snoRNA.** Total RNA was extracted from cells carrying the TB9Cs2C5 snoRNAi silencing construct without induction (-Tet) or after tetracycline induction for 3 days (+Tet), using standard TRIzol protocol, and was subjected to Northern blotting with gene-specific RNA probes. The U3 snoRNA level was used as a loading control**. (iv) Primer extension analysis of Nm on sr1.** Total RNA (8μg) from TB9Cs2C5 (lanes 1-4) and *Nop1* silenced cells (lanes 5-6), before (-Tet) or after 3 days of silencing (+Tet) was subjected to primer extension (see Supplementary table S1 for primers) at low and high dNTP concentrations (and 0.004 mM and 1 mM). Extension products were separated on 8% polyacrylamide–7 M urea gels along with a dideoxynucleotide sequence ladder of the sr1 produced using the same primer. Partial

DNA sequences are given. The potential methylated site is indicated by box**.** The arrow marks a primer extension stop at potential Nm site indicating structural stop. The percentage of band intensity corresponding to RT stop at G126 on the sr1 in the TB9Cs2C5 and *NOP1* silenced cells (compare lane 2, - Tet to lane 4 and 6, + Tet) is given at the right. **(v) Precursor rRNA accumulation following the silencing of TB9Cs2C5 snoRNA**. Total RNA extracted from both un-induced (-Tet) and induced (+Tet) cells following 3 days of silencing was subjected to Northern blotting with indicated ITS probes. The 7SL RNA was used as a loading control **(B) (i) RNA walk" analysis of TB9Cs3C3.** Analysis was performed as in panel A (i) but the RNA was subjected to affinity selection with anti-sense biotinylated oligonucleotide complementary to TB9Cs3C3 snoRNA. The interactions at the rRNA sites for TB9Cs3C3 snoRNA are indicated. **(ii) Identification of C/D snoRNA TB9Cs3C3-precursor rRNA chimeric species.** Chimeric RNA was generated as described in Materials and Methods. RNA from control (-UV) and from the cross-linking experiment (+UV) was subjected to RT-PCR analysis with specific primers (Supplementary Table S1) to both RNA molecules (TB9Cs3C3 snoRNA and pre-rRNA regions are indicated). For other details see legend to panel A (ii). (**iii**) Silencing of TB9Cs3C3 snoRNA. Total RNA was extracted from cells carrying the TB9Cs3C3 snoRNAi silencing construct without induction (-Tet) or after tetracycline induction for 3 days (+Tet), using standard TRIzol protocol, and was subjected to Northern blotting with gene-specific RNA probes. The U3 snoRNA level was used as a loading control. **(iv)** Precursor rRNA accumulation following the silencing of TB9Cs3C3 snoRNA. Total RNA prepared as in panel (iii) and analyzed by northern blot with indicated ITS probes.

Supplementary Figure S3

(A) (i) RNA walk" analysis of TB10Cs4C3. Analysis was performed as in Supplementary Figure 2A (i) but the RNA was subjected to affinity selection with anti-sense biotinylated oligonucleotide complementary to TB10Cs4C3 snoRNA. The significant interactions detected for TB10Cs4C3 snoRNA are indicated in red**. (ii) Identification of C/D snoRNA TB10Cs4C3-precursor rRNA chimeric species.** Chimeric RNA was generated as described in Materials and Methods. RNA from control (-UV) and from the cross-linking experiment (+UV) was subjected to RT-PCR analysis with specific primers (Supplementary Table S1) to both RNA molecules (TB10Cs4C3 snoRNA and pre-rRNA regions are indicated). For other details see figure legend to Supplementary Figure S2A (ii).

(iii) Silencing of TB10Cs4C3 snoRNA. Total RNA was extracted from cells carrying the TB10Cs4C3 snoRNAi silencing construct without induction (-Tet) or after tetracycline induction for 3 days (+Tet), using standard TRIzol protocol, and was subjected to Northern blotting with gene-specific RNA probes. The U3 snoRNA level was used as a loading control.

(iv) Precursor rRNA accumulation following the silencing of TB10Cs4C3 snoRNA. Total RNA prepared as in panel (iii) and analyzed by northern blot with indicated ITS probes.

(B) (i) RNA walk" analysis of TB11Cs3C2. Analysis was performed as in Supplementary Figure 2A (i) but the RNA was subjected to affinity selection with anti-sense biotinylated oligonucleotide complementary to TB11Cs3C2 snoRNA. The interactions at the rRNA sites for TB11Cs3C2 snoRNA are indicated in red. (**ii) Identification of C/D snoRNA TB11Cs3C2-precursor rRNA chimeric species.** Chimeric RNA was generated as described in Materials and Methods. RNA from control (-UV) and from the cross-linking experiment (+UV) was subjected to RT-PCR analysis with specific primers (Supplementary Table S1) to both RNA molecules (TB11Cs3C2 snoRNA and pre-rRNA regions are indicated). For other details see legend to Supplementary Figure S2A (ii).

(iii) Silencing of TB11Cs3C2 snoRNA. Total RNA was extracted from cells carrying the TB11Cs3C2 snoRNAi silencing construct without induction (-Tet) or after tetracycline induction for 3 days (+Tet), using standard TRIzol protocol, and was subjected to Northern blotting with gene-specific RNA probes. The U3 snoRNA level was used as a loading control. (iv) **Precursor rRNA accumulation following the silencing of TB11Cs3C2 snoRNA**. Total RNA prepared as in panel (iii) and analyzed by northern blot with indicated ITS probes.

Supplementary Figure S4

(A) (i) "RNA walk" analysis of TB10Cs1C4. Analysis was performed as in Supplementary Figure S2A (i) but the RNA was subjected to affinity selection with anti-sense biotinylated oligonucleotide complementary to TB10Cs1C4 snoRNA. The significant interactions detected for TB10Cs1C4 snoRNA are indicated in red. **(ii) Identification of C/D snoRNA TB10Cs1C4-precursor rRNA chimeric species.** Chimeric RNA was generated as described in Materials and Methods. RNA from control (-UV) and from the cross-linking experiment (+UV) was subjected to RT-PCR analysis with specific primers (Supplementary Table S1) to both RNA molecules (TB10Cs1C4 snoRNA and pre-rRNA regions are indicated). For other details see figure legend to Supplementary Figure S2A (ii). **(iii) Silencing of TB10Cs1C4 snoRNA.** Total RNA was extracted from cells carrying the TB10Cs1C4 snoRNAi silencing construct without induction (-Tet) or after tetracycline induction for 3 days (+Tet), using standard TRIzol protocol, and was subjected to Northern blotting with gene-specific RNA probes. The U3 snoRNA level was used as a loading control. **(iv) Precursor rRNA accumulation following the silencing of TB10Cs1C4 snoRNA**. Total RNA was extracted from *T. brucei* cells carrying TB10Cs1C4 snoRNAi construct before and after 3 days of Tetracycline induction. RNA run on agarose-formaldehyde gel and northern blots were hybridized with indicated ITS probes.

(B) (i) RNA walk" analysis of TB10Cs1C1. Analysis was performed as in Supplementary Figure 2A (i) but the RNA was subjected to affinity selection with anti-sense biotinylated oligonucleotide complementary to TB10Cs1C1 snoRNA. The interactions at the rRNA sites for TB10Cs1C1 snoRNA are indicated in red**. (ii) Identification of C/D snoRNA TB10Cs1C1-precursor rRNA chimeric species.** Chimeric RNA was generated as described in Materials and Methods. RNA from control (-UV) and from the cross-linking experiment (+UV) was subjected to RT-PCR analysis with specific primers (Supplementary Table S1) to both RNA molecules (TB10Cs1C1 snoRNA and pre-rRNA regions are indicated). For other details see legend to Supplementary Figure S2A (ii). **(iii) Silencing of TB10Cs1C1 snoRNA.** Total RNA was extracted from cells carrying the TB10Cs1C1 snoRNAi silencing construct without induction (-Tet) or after tetracycline induction for 3 days (+Tet), using standard TRIzol protocol, and was subjected to Northern blotting with gene-specific RNA probes. The U3 snoRNA level was used as a loading control. **(iv) Precursor rRNA accumulation following the silencing of TB10Cs1C1 snoRNA**. Total RNA prepared as in panel (iii) and analyzed by northern blot with ITS2 and ITS7 probes.

Supplementary Figure S5

Silencing of snoRNA and Northern blot analysis of precursor rRNA species in snoRNA silenced cells.

(A) Silencing of snoRNA. Total RNA was prepared from cells carrying the specific snoRNAi silencing construct without induction (-Tet) or after tetracycline induction for 3 days (+Tet), using standard TRIzol protocol, and was subjected to Northern blotting with gene-specific RNA probes. The U3 snoRNA level was used as a loading control. (i) TB8Cs1C1; (ii) TB8Cs1C3; (iii) TB9Cs2C3; (iv) TB9Cs3H2.

(B) Northern blotting. Total RNA extracted from both un-induced (-Tet) and induced (+Tet) snoRNAi cells following 3 days of silencing and run on agarose- formaldehyde gel electrophoresis. Northern blots were hybridized with ITS2 and ITS7 probes. The 7SL RNA was used as a loading control. (i) TB8Cs1C1; (ii) TB8Cs1C3; (iii) TB9Cs2C3; (iv) TB9Cs3H2.

Supplementary Figure S6

Nascent RNA synthesis in permeable cells before and after silencing of TB9Cs2C5 snoRNA. Permeable cells were prepared from $2x10^8$ cells carrying the TB9Cs2C5 snoRNAi silencing construct, without induction (-Tet) or after tetracycline induction for 3 days (+Tet), as described in the Methods. Permeable trypanosomes were pre-treated with 100 µg/ml α-amanitin. The RNA was fractionated by electrophoresis on a 6% polyacrylamide-7 M urea gel. The identity of the various RNA species is indicated by arrows. The location of nascent transcribed RNAs is indicated by dashed line boxes. Lanes 1, 2, 3- variable amounts of RNA from uninduced cells; lanes 4, 5, 6- variable amounts of RNA from silenced cells. Long exposure (*left* panel), short exposure (*right* panel).

Supplementary Figure S7

snoRNA interfering with ribosome biogenesis mediates rRNA modifications in the ribosome decoding center.

Two snoRNA species reported in this study mediate modifications to SSU locations that reside within the SSU decoding center. These modifications are in close proximity to eL41, a LSU ribosomal protein that serves as a eukaryote specific ribosomal bridge. Modified residues are highlighted in magenta and are represented as spheres; eL41 is presented by a

green surface, tRNAs in yellow. Residue numbers in *T. brucei* are indicated, with snoRNA numbering in parenthesis.

REFERENCES

1. Michaeli,S., Doniger,T., Gupta,S.K., Wurtzel,O., Romano,M., Visnovezky,D., Sorek,R., Unger,R. and Ullu,E. (2012) RNA-seq analysis of small RNPs in Trypanosoma brucei reveals a rich repertoire of non-coding RNAs. *Nucleic Acids Res.*, **40**, 1282–1298.
2. Barth,S., Shalem,B., Hury,A., Tkacz,I.D., Liang,X.-H., Uliel,S., Myslyuk,I., Doniger,T., Salmon-Divon,M., Unger,R., *et al.* (2008) Elucidating the role of C/D snoRNA in rRNA processing and modification in Trypanosoma brucei. *Eukaryot. Cell*, **7**, 86–101.
3. Gupta,S.K., Hury,A., Ziporen,Y., Shi,H., Ullu,E. and Michaeli,S. (2010) Small nucleolar RNA interference in Trypanosoma brucei: mechanism and utilization for elucidating the function of snoRNAs. *Nucleic Acids Res.*, **38**, 7236–47.
