# Supplementary figures and images for: Small nucleolar RNAs controlling rRNA processing in Trypanosoma brucei
Source: Nucleic Acids Res. 2019 Jan 3;47(5):2609–29. doi: 10.1093/nar/gky1287 (PMC6411936; doi:10.1093/nar/gky1287)

Supplementary Figure S5

A

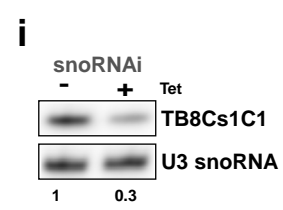

B

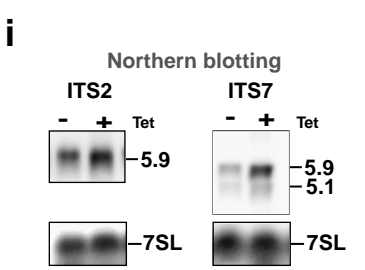

**ii**

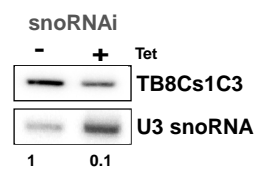

**ii**

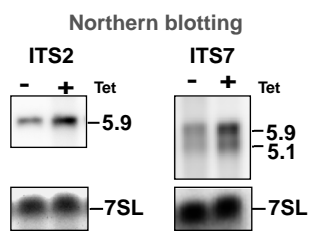

**iii**

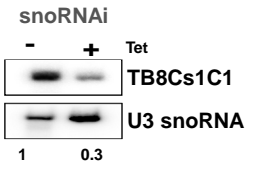

**iii**

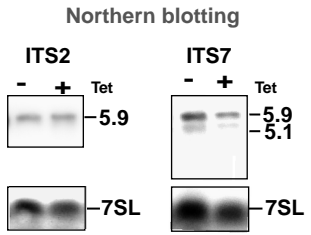

**iv**

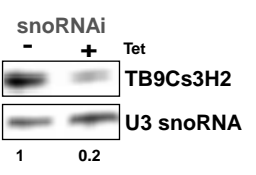

**iv**

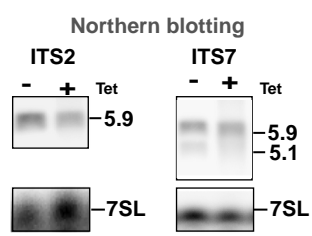

Supplement: Supplementary Data [file gky1287_supplemental_files.zip › Chikne et al Supplementary Figure S5.pdf]

Supplementary Figure S6

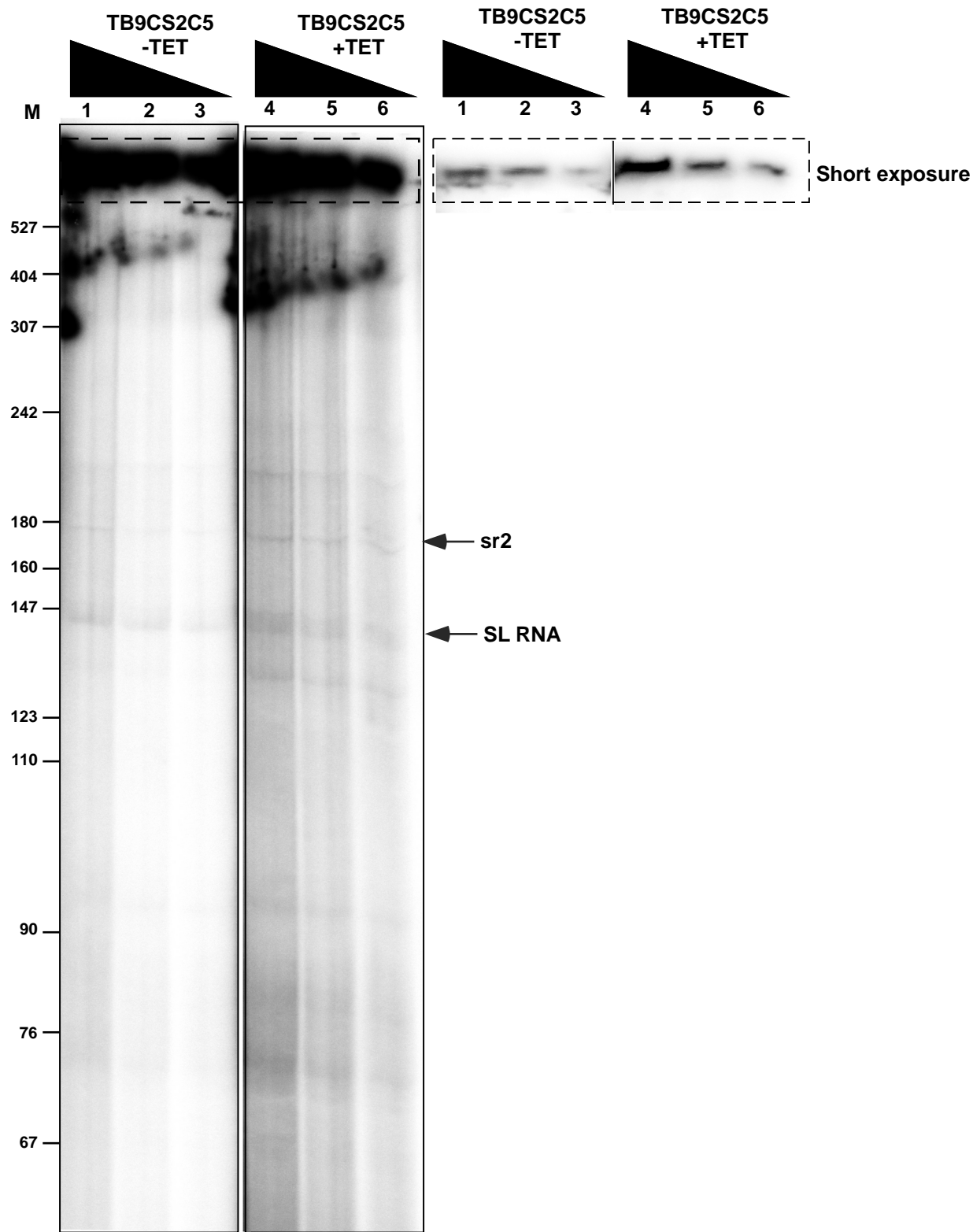

Supplement: Supplementary Data [file gky1287_supplemental_files.zip › Chikne et al Supplementary Figure S6.pdf]

## Supplementary Figure S7

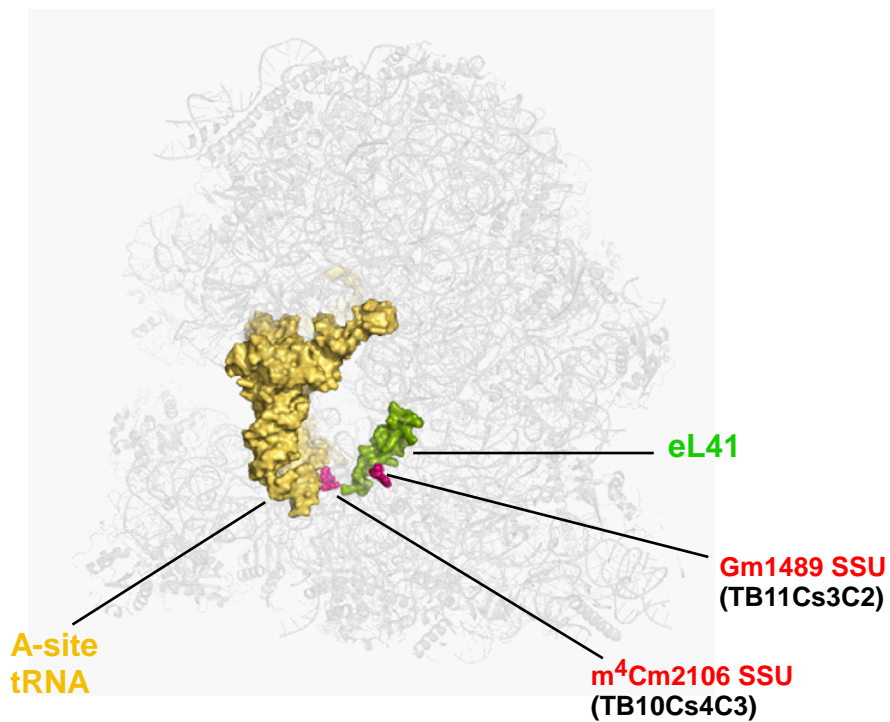

Supplement: Supplementary Data [file gky1287_supplemental_files.zip › Chikne et al Supplementary Figure S7.pdf]

# Supplementary Figure S1

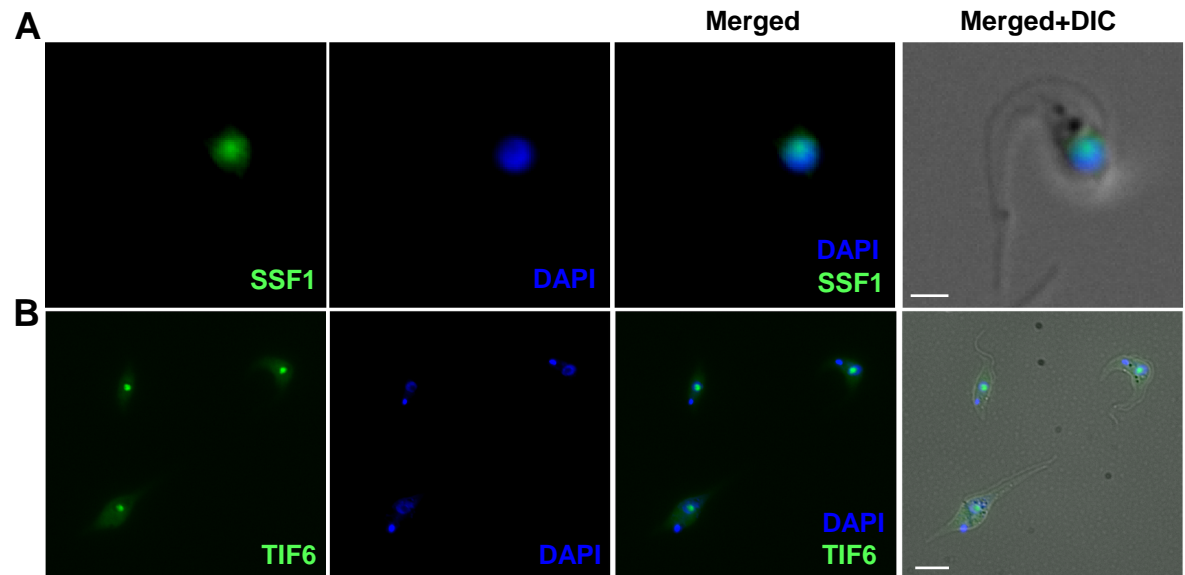

Supplement: Supplementary Data [file gky1287_supplemental_files.zip › Chikne et al_Supplementary Figure S1.pdf]
